# A growth reference for mid upper arm circumference for age among school age children and adolescents, and validation for mortality: growth curve construction and longitudinal cohort study

**DOI:** 10.1136/bmj.j3423

**Published:** 2017-08-03

**Authors:** Lazarus Mramba, Moses Ngari, Martha Mwangome, Lilian Muchai, Evasius Bauni, A Sarah Walker, Diana M Gibb, Gregory Fegan, James A Berkley

**Affiliations:** 1 Department of Medicine, University of Florida, FL, USA; 2 KEMRI/Wellcome Trust Research Programme, PO Box 230-80108, Kilifi, Kenya; 3 The Childhood Acute Illness & Nutrition (CHAIN) Network, Nairobi, Kenya; 4 Jomo Kenyatta University of Agriculture and Technology, Nairobi, Kenya; 5 MRC Clinical Trials Unit, University College London, London, UK; 6 Nuffield Department of Medicine, University of Oxford, Oxford, UK; 7 Swansea Trials Unit, Swansea University Medical School, Swansea, UK

## Abstract

**Objectives** To construct growth curves for mid-upper-arm circumference (MUAC)-for-age z score for 5-19 year olds that accord with the World Health Organization growth standards, and to evaluate their discriminatory performance for subsequent mortality.

**Design** Growth curve construction and longitudinal cohort study.

**Setting** United States and international growth data, and cohorts in Kenya, Uganda, and Zimbabwe.

**Participants** The Health Examination Survey (HES)/National Health and Nutrition Examination Survey (NHANES) US population datasets (age 5-25 years), which were used to construct the 2007 WHO growth reference for body mass index in this age group, were merged with an imputed dataset matching the distribution of the WHO 2006 growth standards age 2-6 years. Validation data were from 685 HIV infected children aged 5-17 years participating in the Antiretroviral Research for Watoto (ARROW) trial in Uganda and Zimbabwe; and 1741 children aged 5-13 years discharged from a rural Kenyan hospital (3.8% HIV infected). Both cohorts were followed-up for survival during one year.

**Main outcome measures** Concordance with WHO 2006 growth standards at age 60 months and survival during one year according to MUAC-for-age and body mass index-for-age z scores.

**Results** The new growth curves transitioned smoothly with WHO growth standards at age 5 years. MUAC-for-age z scores of −2 to −3 and less than−3, compared with −2 or more, was associated with hazard ratios for death within one year of 3.63 (95% confidence interval 0.90 to 14.7; P=0.07) and 11.1 (3.40 to 36.0; P<0.001), respectively, among ARROW trial participants; and 2.22 (1.01 to 4.9; P=0.04) and 5.15 (2.49 to 10.7; P<0.001), respectively, among Kenyan children after discharge from hospital. The AUCs for MUAC-for-age and body mass index-for-age z scores for discriminating subsequent mortality were 0.81 (95% confidence interval 0.70 to 0.92) and 0.75 (0.63 to 0.86) in the ARROW trial (absolute difference 0.06, 95% confidence interval −0.032 to 0.16; P=0.2) and 0.73 (0.65 to 0.80) and 0.58 (0.49 to 0.67), respectively, in Kenya (absolute difference in AUC 0.15, 0.07 to 0.23; P=0.0002).

**Conclusions** The MUAC-for-age z score is at least as effective as the body mass index-for-age z score for assessing mortality risks associated with undernutrition among African school aged children and adolescents. MUAC can provide simplified screening and diagnosis within nutrition and HIV programmes, and in research.

## Introduction

In many parts of the world, school age children and adolescents are vulnerable to food insecurity, conflict, and natural disasters. For example, in Syria, three million children are in camps,^1^ ^2^ and in Eastern Kenya, the United Nations estimated that 156 000 Somali school age children were refugees in the Dadaab camps.^3^ Endemic undernutrition among school age children is also widespread in both rural and urban areas in non-crisis situations in low income countries.^4^ ^5^ Throughout developing countries, as a result of earlier screening and effective antiretroviral drugs, increasing numbers of children with HIV infection are surviving into adolescence, more than 80% of whom are estimated to live in sub-Saharan Africa.^6^ HIV is commonly accompanied by malnutrition because of infections, inflammation, enteropathy, anorexia, increased household food insecurity, and orphanhood.^7^ ^8^

The World Health Organization recommends using body mass index to assess malnutrition in school aged children, adolescents, and adults. In 2007, WHO published growth references for weight, height, and body mass index for 5-19 year olds using historical data from the United States merged with prospective data from the 2006 WHO Multicentre Growth Reference Study of under 5s, and growth curves modelled using the statistical methods developed for that study.^9^

Among under 5s, mid upper arm circumference (MUAC) is the mainstay of identification of malnutrition in the community and increasingly used in health facilities. Measuring MUAC is much cheaper and easier than measuring weight and height, is less affected by acute dehydration than weight based indices,^10^ and is a better predictor of survival than weight-for-height z score.^11^ ^12^ ^13^ MUAC was not, however, included in the 2007 WHO growth references for 5-19 year olds. A MUAC reference for US children and adolescents has recently been published, but at age 5 years, z score values from −3 to 3 are between 0.6 cm and 2.6 cm higher than those of the 2006 WHO growth standards.^14^ Thus there is currently no internationally accepted reference, nor any studies that relates either MUAC or body mass index to subsequent major health outcomes in this age group.^15^

We considered that MUAC may be a useful screening and diagnostic tool for undernutrition among school aged children and adolescents in situations of food insecurity, at health facilities, and in HIV programmes. We created MUAC-for-age z score growth references for 5-19 year olds that accord with the WHO 2006 growth standards for children under 5 years old.^16^ We used the same datasets and similar methods that were used by WHO to create the 2007 growth references for body mass index. To validate the new growth references, we evaluated the predictive value of both MUAC-for-age and body mass index-for-age z scores for subsequent mortality in two longitudinal cohorts in Africa.

## Methods

### Preparation of datasets

We obtained publically available data from www.cdc.gov/nchs/data_access/ftp_data.htm (MUAC is described here as upper arm girth). These datasets were based on a US nationwide probability sample, selected so that certain population groups thought to be at risk of malnutrition (people on low incomes, preschool children, women of childbearing age, and elderly people) were oversampled at preset rates. The Health Examination Survey (HES) cycles II and III, and the National Health and Nutrition Examination Survey (NHANES) cycle I included data from 7119 children aged 6-11 years, 6768 children aged 12-17 years, and 23 808 people aged 1-74 years, respectively. For the HES datasets, all observations were initially included. For NHANES cycle I, we included observations from young people aged 5-25 years. These datasets were merged, giving a total number of observations from 20 953 individuals then stratified by sex (10 639 females). We fitted generalized additive models for location, scale, and shape (GAMLSS) and excluded measurements outside 4 standard deviations. This method was chosen, rather than excluding below −3 SD and above 2 SD, which was done by WHO in 2007, so as not to exclude a small number of biologically plausible measures in the outer centiles.^17^

To achieve a smooth transition with the WHO growth standards at age 60 months, we imputed normally distributed z scores for 36 000 hypothetical individuals, randomly assigned by sex (17 914 girls and 18 086 boys) and by age, uniformly distributed from 24-71 months. We then used the MUAC L, M, and S coefficients from the WHO growth standards^16^ (with additional summary data from the Multicentre Growth Reference Study for 60-71 months obtained from M de Onis at WHO, personal communication, 2014) to back transform the z scores to MUAC measurements. The imputed datasets for girls and boys were then merged with the cleaned HES/NHANES dataset.

### Statistical analysis

We modeled MUAC, stratified by sex, as a function of age by fitting GAMLSS models, testing different transformations, including Box-Cox-Power-Exponential, Box-Cox-t, and Box-Cox-Cole-Green models.^18^ ^19^ ^20^ The Box-Cox-Cole-Green models provided the closest transition with the WHO 2006 standards. Box-Cox-Power-Exponential and Box-Cox-t models had similar performance by Akaike Information Criteria with no practical differences in the outer centiles over Box-Cox-Cole-Green models, suggesting that it was not necessary to model kurtosis (see supplementary table 1). We used penalized B-splines to smooth the z scores and centile curves to reduce irregularities that tend to occur irrespective of the size of a dataset from sampling and measurement variability.^9^ ^21^ ^22^ To reduce edge effects we then truncated the results at ages 5 and 19 years. All of these analyses were conducted using the GAMLSS package^22^ within the R statistical environment.^23^

### Validation for mortality

The validity of anthropometry for public health applications is optimally assessed by its predictive value for subsequent mortality.^12^ ^24^ ^25^ ^26^

Firstly, we examined the discriminatory value of MUAC-for-age and body mass index-for-age z scores for subsequent mortality during one year among all of the HIV infected children aged 5-17 years who were enrolled between 2007 and 2008 into the Antiretroviral Research for Watoto (ARROW) clinical trial in HIV-1 infected children in Uganda and Zimbabwe (www.controlled-trials.com/ISRCTN24791884).^27^ Children were enrolled at the time of starting antiretroviral treatment. We measured MUAC, weight, and height at baseline, and recorded the dates of death or loss to follow-up. Ready to use therapeutic food was only provided to children under 6 years of age at the study sites.

Secondly, we examined mortality during one year after discharge from hospital among children aged 5-13 years residing within the Kilifi Health and Demographic Surveillance System (KHDSS) in rural Kenya^28^ who had been consecutively admitted to Kilifi County Hospital 2007-2012. When multiple admissions were recorded, we only used the first admission in the analysis. Anthropometry was routinely undertaken and recorded at admission, and provider initiated HIV testing was offered. We determined mortality during the following year through the KHDSS quarterly household census. Nutrition services at the time were targeted to children under 5 years old. Comprehensive care for HIV was provided at the hospital. In a previous study in Kilifi in 2004-08, the mortality rate among children aged 0-14 years (predominantly under 5s) discharged from hospital was 7.7 times greater than among children in the community, and it was strongly associated with weight for age.^29^

Using the 2007 WHO growth reference, we calculated MUAC-for-age z scores for children in the validation datasets from the new growth reference and body mass index-for-age z scores. We estimated hazard ratios for death using Cox proportional hazard models for predefined categories of MUAC-for-age and body mass index-for-age z scores (less than −3, −3 to −2, and −2 or more, plus “missing” as a separate category). The proportions of children identified as malnourished by these MUAC-for-age and body mass index-for-age thresholds were compared using a McNemar test. Multivariable models included age, sex, and HIV status (for the Kenyan dataset) as a priori potential confounders. We treated a missing or declined HIV test as a separate category, as declined tests were not assumed to have occurred randomly. Children in the ARROW trial were all HIV infected and so we adjusted multivariable models for age and sex only. To evaluate the predictive value of continuous MUAC-for-age and body mass index-for-age z scores, we estimated the area under the receiver operating characteristic curves (AUC) for death within one year, and tested differences in AUC using the method of DeLong et al.^30^

In exploratory analyses of the validation datasets, we examined the discriminatory value for mortality of MUAC-for-age and body mass index-for-age z scores by age and sex, including likelihood ratio tests for interaction within logistic regression models (using ARROW trial data with an age range 5 to 17 years); and the performance of simplified cut-offs for MUAC derived from a linear increment by year of age from the WHO cut-off of 12.5 cm in under 5s to 21 cm at age 19 years, which is a cut-off commonly used for adults.^15^

### Participant involvement

The need for a more simple tool such as MUAC to assess undernutrition among school-aged children and adolescents, and design of the study were informed by discussions with professional and lay people involved in hospital care, HIV care, and nutrition services; policy makers; individuals working with international humanitarian agencies; and through online discussion forums (including www.en-net.org/question/1744.aspx, www.ennonline.net/fex/54/cutoffpointsadolescentssyria, www.en-net.org/question/1832.aspx, www.en-net.org/question/522.aspx). HES and NHANES and the WHO Multicentre Growth Reference Study were undertaken with the informed intent that participation would contribute to the development of health and growth references as a benefit to society. The validation utilized data already collected. The ARROW trial was conducted in the context of an active community engagement process, and is one of a series of clinical trials concerning the treatment of HIV involving a wider stakeholder group, through which findings are disseminated. The KEMRI/Wellcome Trust Research Programme undertakes continuous community engagement through regular meetings with the community involving KEMRI-community representatives where research projects are discussed, feedback from the community sought, and results disseminated.

## Results

### Growth references

Figures 1 and 2 show the new MUAC-for-age z score growth curves by sex (see supplementary file for tables of z scores and centiles and supplementary figures showing reference curves for girls and boys for clinical and programmatic use). At age 60 months, the MUAC-for-age z values for boys and girls were closely aligned with the existing WHO growth standards, within 1 mm either way for z scores −3 to +2, and 3 mm (boys)/2 mm (girls) at z score +3 (fig 3). Up to 14 years of age, boys and girls followed approximately similar trajectories. After 14 years, boys continued to grow at a faster rate than girls. Hence, at age 19 years z scores of −2 and −3 representing moderate and severe undernutrition were 21.3 cm and 19.6 cm for women, and higher for men at 24.6 cm and 22.7 cm.

**Figure f1:**
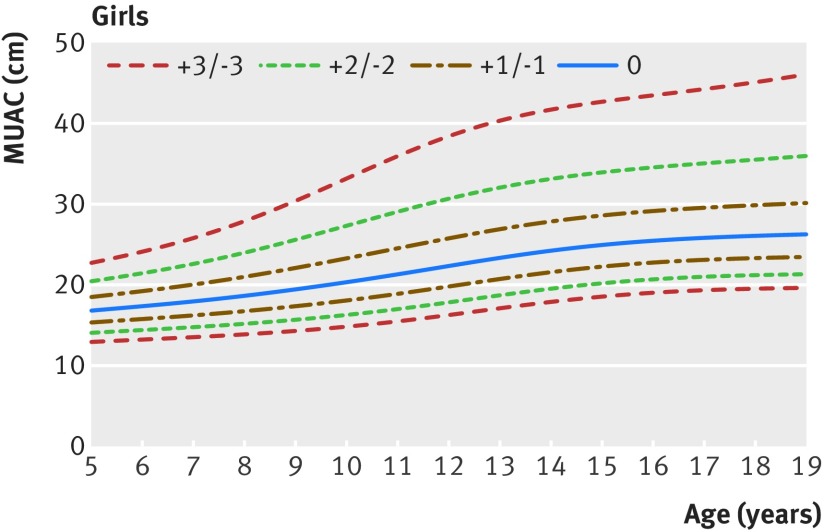
**Fig 1** Mid upper arm circumference (MUAC)-for-age z score reference curves for girls aged 5 to 19 years

**Figure f2:**
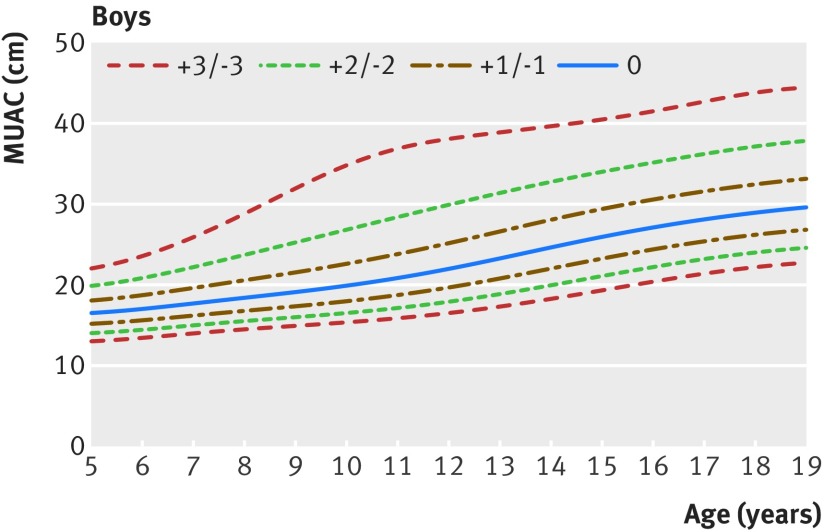
**Fig 2** Mid upper arm circumference (MUAC)-for-age z score reference curves for boys aged 5 to 19 years

**Figure f3:**
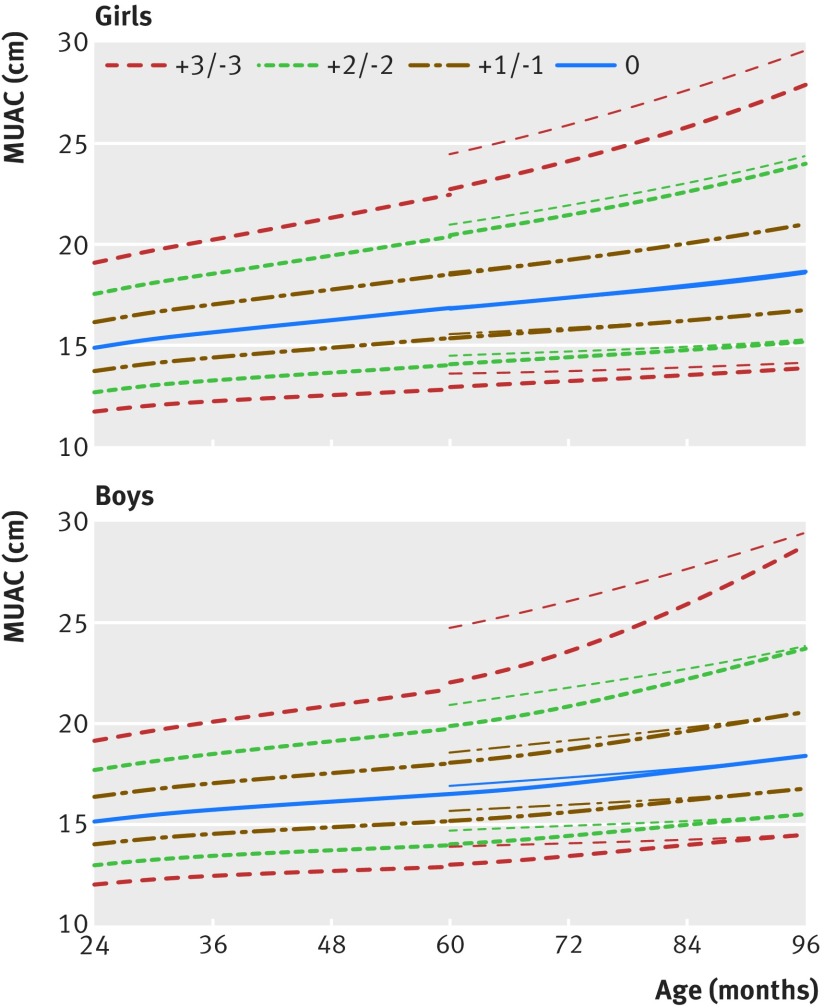
**Fig 3** Transition from World Health Organization (2006) standards for under 5s to mid upper arm circumference (MUAC)-for-age z scores for 5 to 19 year olds

### Validation

We used data from 685 HIV infected children who were aged 5-17 years (median 8.8 years) at enrolment into the ARROW trial in Uganda and Zimbabwe between January 2007 and October 2008. Their mean weight for age, height for age, body mass index, and MUAC z scores at enrolment were −1.9 (SD 1.1), −2.1 (SD 1.3), −0.9 (SD 1.2), and −1.7 (SD 1.6), respectively. No values were missing for baseline anthropometry or outcomes. MUAC-for-age z scores of less than −2 and less than −3 were observed in 218 (32%) and 100 (15%) children, respectively (table 1). A smaller proportion of participants would have been classified as undernourished by body mass index-for-age z score than by MUAC-for-age z score: 102 (15%) were less than −2 z scores and 44 (6.4%) less than −3 z scores for body mass index-for-age z scores (P<0.001).

**Table 1 tbl1:** Hazard ratios for mortality within one year of enrolment into the Antiretroviral Research for Watoto (ARROW trial), according to mid upper arm circumference (MUAC)-for-age and body mass index (BMI)-for-age z score categories (5 to 17 years old)

z scores	No	Died	Hazard ratio (95% CI)	P value	Adjusted hazard ratio* (95% CI)	P value
MUAC-for-age:						
−2 or more	467	4	1	-	1	-
−3 to −2	118	4	4.0 (1.00 to 16.0)	0.05	3.63 (0.90 to 14.7)	0.07
Less than −3	100	10	12.2 (3.84 to 39.0)	<0.001	11.1 (3.40 to 36.0)	<0.001
BMI-for-age:						
−2 or more	583	9	1	-	1	-
−3 to −2	58	2	2.22 (0.48 to 10.3)	0.3	1.91 (0.41 to 8.91)	0.4
Less than −3	44	7	11.1 (4.15 to 30.0)	<0.001	9.34 (3.42 to 25.5)	<0.001

*Adjusted for age and sex.

Overall, 18 (2.6%) children died within one year, a rate of 25 deaths (95% confidence interval 16 to 41 deaths) per 1000 child years during 674 child years of observation. Four deaths (22%) occurred in children without undernutrition (both MUAC-for-age z scores and body mass index-for-age z scores of –2 or more, see supplementary table 2). MUAC-for-age and body mass index-for-age z scores of less than −3 were both independently associated with mortality (table 1). The sensitivities for death at a cut-off at less than −2 z scores were 78% (95% confidence interval 52% to 94%) for MUAC-for-age z scores, and 50% (26% to 74%) for body mass index-for-age z scores. However, there was no evidence of a difference between the AUCs of MUAC-for-age and body mass index-for age z scores for mortality: 0.81 (95% confidence interval 0.70 to 0.92) and 0.75 (0.63 to 0.86, respectively; absolute difference in AUC 0.06 (95% confidence interval −0.032 to 0.16, P=0.2).

In exploratory subgroup analyses, the point estimates of AUC for both MUAC-for-age and body mass index-for-age z scores were higher among children aged 10-17 years (see supplementary table 3), but differences and tests for interaction between MUAC-for-age z score and age (P=0.2) or sex (P=0.07) were not statistically significant. Sex independent, simplified cut-offs for age in completed years were generated (see supplementary table 3 and supplementary figure 7) and their discriminatory performance evaluated. The simplified linear MUAC cut-off identified 164 (24%) participants, with sensitivity for death of 72% (95% confidence interval 47% to 90%), and the AUC was not statistically different from cut-offs at MUAC-for-age z scores less than −2 or body mass index-for-age z scores less than −2 (see supplementary table 4 and supplementary figure 8).

In Kenya, 1741 children aged 5-13 years (median 7.4 years) resident in the Kilifi Health and Demographic Surveillance System area were discharged alive after admission to Kilifi County Hospital between January 2007 and December 2012. Eight children were missing measurements for MUAC, 42 for body mass index, and 53 for follow-up outcome. The mean MUAC-for-age and body mass index-for-age z scores were −1.9 (SD 1.4) and −1.4 (SD 1.4), respectively. The MUAC-for-age z score was less than −2 in 700 (40%) children and less than −3 in 275 (16%) children, whereas the body mass index-for-age z score was less than −2 in 443 (25%) children and less than −3 in 166 (9.5%) children (P<0.001) (table 2). Sixty six (3.8%) children were infected with HIV, and 145 (8.3%) had a missing or declined HIV test.

**Table 2 tbl2:** Hazard ratios for mortality within one year of discharge from Kilifi District Hospital, Kenya according to mid upper arm circumference (MUAC)-for-age and body mass index (BMI)-for-age z score categories (5 to 13 year olds)

z scores	No*	Died	Hazard ratio (95% CI)	P value	Adjusted hazard ratio† (95% CI)	P value
MUAC-for-age:						
−2 or more	959	13	1	-	1	-
−3 to −2	435	13	2.26 (1.03 to 4.96)	0.04	2.22 (1.01 to 4.89)	0.04
Less than −3	286	20	5.92 (2.94 to 11.9)	<0.001	5.15 (2.49 to 10.7)	<0.001
BMI-for-age:						
−2 or more	1213	28	1	-	1	-
−3 to −2	272	6	0.97 (0.40 to 2.34)	0.9	0.91 (0.37 to 2.20)	0.8
Less than −3	163	12	3.29 (1.67 to 6.47)	<0.001	2.92 (1.47 to 5.84)	0.002

*53 children with missing outcome, eight with missing MUAC, and 42 with missing BMI are excluded from this table (there were no deaths among children with missing MUAC or BMI, but two children missing BMI were also missing outcome).

†Adjusted for age, sex, and HIV status.

Overall, 46 (2.6%) children died within one year after discharge during 1648 child years of observation, giving a rate of 28 deaths (95% confidence interval 21 to 37 deaths) per 1000 child years. No deaths occurred among children with missing MUAC or body mass index measurements. Twelve deaths (26%) occurred in children without undernutrition (both MUAC-for-age and body mass index-for-age z scores were –2 or more) (see supplementary table 3). The sensitivities for death at a cut-off of less than −2 z scores were 72% (95% confidence interval 57% to 84%) for MUAC-for-age z score, and 39% (25% to 55%) for body mass index-for-age z score. Hazard ratios for both indices were only slightly attenuated by adjustment for age and HIV status (table 2). The AUC for mortality within one year was greater for MUAC-for-age z scores (0.73, 95% confidence interval 0.65 to 0.80) than for body mass index-for-age z scores (0.58, 0.49 to 0.67); absolute difference in AUC 0.15 (95% confidence interval 0.073 to 0.23; P=0.0002).

In exploratory subgroup analyses, the AUCs for MUAC-for-age z scores were higher than for body mass index-for-age z scores among both boys and girls (see supplementary table 7). Simplified MUAC cut-offs identified 488 (29%) of participants, sensitivity for death of 50% (95% confidence interval 35% to 65%), and an AUC not statistically different from cut-offs of MUAC-for-age z scores less than −2 or body mass index-for-age z scores less than −2, but lower than for continuous MUAC-for-age z scores (see supplementary table 8 and supplementary figure 9).

## Discussion

Inexpensive and simple methods for diagnosing malnutrition can have considerable utility in resource poor settings for guiding admission to feeding programmes or further medical investigation, provided they are valid. We have constructed growth curves for mid upper arm circumference (MUAC) in school age children and adolescents that converge with the 2006 WHO growth standards. Studies have not previously examined associations between body mass index-for-age z scores, MUAC-for-age z scores, or any other anthropometric measure and mortality among school age children and adolescents.**]** In two separate prospective cohorts, we found that MUAC-for-age z score was at least as good as the existing body mass index-for-age z score reference at discriminating subsequent mortality. As with body mass index or weight for height, MUAC cannot distinguish primary malnutrition from other causes; however, it is an effective marker of risk.

The predictive value of both MUAC and body mass index, assessed by AUC, was greater among HIV infected children in the ARROW trial, most likely because of less heterogeneity than in the Kenyan cohort, where a greater proportion of deaths occurred among individuals without low anthropometric values. Using a z score threshold of −3, MUAC classified a greater number of children as being severely malnourished than did body mass index, which may have programmatic implications, but reflects a higher sensitivity for mortality. The determination of thresholds for interventions may also depend on local context and resources and the costs, potential adverse effects, and efficacy of the specific interventions.

### Interpretation of MUAC

Previous studies in this age group have reported a close correlation between MUAC and body mass index values, and that MUAC is more aligned with fat mass than with fat-free mass.^31^ ^32^ ^33^ ^34^ However, in a trial of meat supplementation in schoolchildren in Kenya, the rapid increase in MUAC observed in the group allocated to meat reflected increased arm muscle area rather than increased arm fat.^35^ Among adolescent girls in Mozambique, MUAC correlated with levels of haemoglobin, serum albumin, ferritin, zinc, and plasma retinol.^36^ For predicting mortality among adults in a famine setting, the AUC was statistically significantly greater for MUAC (0.71) than for body mass index (0.57).^37^

In this study we did not aim to examine the ability of MUAC to detect overweight and obesity, and these were rare in our validation datasets. However, several studies, including one comprising data from 12 countries, report a close correlation between MUAC and body mass index in this age group, likely due to its concordance with fat mass,^31^ ^32^ ^33^ ^34^ and have suggested diagnostic cut-off values.^31^ ^33^ ^34^ ^38^ ^39^ Besides simply examining correlations with body mass index, ideally studies should validate MUAC in relation to markers of disease or functional outcomes. Among South African and Sardinian children, MUAC was strongly independently correlated with systolic blood pressure.^40^ ^41^ ^42^

### Age and sex dependency of MUAC

Adolescence presents potential difficulties for anthropometric references. All of the commonly used measures are influenced by changes in body proportions and composition and the degree of sexual maturation, which may affect interpretation during the second decade of life.^43^ ^44^ Importantly, undernutrition, inflammation, stress, or illnesses that typically occur in HIV infection or during humanitarian emergencies, may delay puberty.^45^ ^46^ ^47^ ^48^ While adjustment for the stage of sexual maturation and the onset of menarche may be technically desirable, and of value in interpreting an individual’s growth, it may not always be culturally acceptable to evaluate these routinely in the settings where MUAC is likely to be most useful. For individual assessment of growth, changes over time, diet, and context are important factors.^49^

The MUAC-for-age z score growth curves are similar between sexes up to approximately age 14 years, after which boys continued to grow faster than girls. Few studies have assessed MUAC growth in this age group. However, the pattern of higher MUAC values in males aged more than 15 years was observed in all the (non-US) studies that we identified, undertaken in Turkey, India, China, and the UK.^50^ ^51^ ^52^ ^53^ Furthermore, in datasets from five African countries, India, China, and Papua New Guinea,^54^ men had larger MUAC measures than women. Together, these suggest that a sex differential from mid-teens is a generalised phenomenon, rather than a peculiarity of the HES/NHANES dataset. Our exploratory subgroup analysis suggested no decline in prognostic performance of MUAC-for-age z score during later adolescence in either sex, possibly because of the associations between health and delayed puberty.

Another potential concern for age based anthropometric indices is that age may not be accurately known. Adjusting MUAC values for height rather than for age, for example, has been attempted but has not been shown to improve predictive value, despite potential inaccuracies in age.^12^ There are also clinical and humanitarian situations were individuals may be too weak or sick to stand.^49^

In settings of humanitarian assistance or when large numbers of people need to be efficiently assessed, simple sex independent anthropometric thresholds are attractive, but they involve compromises. This is well illustrated by the current fixed MUAC cut-off of 12.5 cm for moderate acute malnutrition between 6 months and 5 years of age. According to the WHO 2006 growth standards, at age 6 months, 12.5 cm is equivalent to MUAC for age z scores of −1.2 and −1.7 in girls and boys, respectively. At age 5 years, the z scores would indicate severe malnutrition, but the sex differences are less, with z scores of −3.3 in girls and −3.4 in boys. However, a fixed cut-off is used operationally because of its simplicity, because younger children have a higher risk of death associated with undernutrition, and because although the sex bias is well known, it is considered acceptable. In exploratory analyses, we found no evidence that a simple rule of a linear progression in MUAC between 12.5 cm in under 5s and 21 cm at age 19 years performed less well than binary cut-offs of MUAC-for-age z scores less than −2 or body mass index-for-age z scores less than −2, but it did perform less well than continuous MUAC-for-age z score. Because of a sex difference in growth in late teens, a sex independent cut-off would similarly introduce a sex bias towards detecting girls at older ages.

### Considerations for a standardised MUAC growth reference

A key consideration for developing these growth curves was the use of a historical US dataset, as was used for the existing WHO growth reference for body mass index in this age group. Differences are likely to exist between US and developing country populations—for example, in relation to pubertal timing and body shape, even though the predominant survey used, NHANES I, was oversampled for groups at risk of malnutrition.^55^ Although the current epidemic of overweight and obesity was already emerging in the early 1970s, this is likely to have influenced the upper z scores rather than the lower ones. The use of the HES/National Center for Health Statistics reference population has been questioned (as previously applied to body mass index, weight, and height, before the 2007 WHO growth references).^49^ Earlier work on international measures of obesity included using pooled historical data on body mass index from Brazil, Great Britain, Hong Kong, the Netherlands, Singapore, and the United States^56^; however, these datasets lacked MUAC measurements. In the absence of prospective longitudinal studies of the growth of optimally healthy reference populations in developing countries, such as was undertaken within the Multicentre Growth Reference Study that generated the standards for children under 5 years old,^16^ the HES/NHANES dataset remains the best currently available option for a standardised MUAC reference. Importantly, it is now shown to be effective at predicting subsequent mortality in this age group in Africa. Use of the HES/NHANES dataset ensures that the different anthropometric references for this age group (weight, height, body mass index, MUAC) are all based on the same underlying population.

### Limitations for validation

A limitation for validation was that our datasets included a limited number of participants in their late teens, and further validation of the discriminatory value of MUAC for mortality among boys and girls in late adolescence in different settings would be valuable.

### Further research

Further research is needed to determine optimal intervention packages to treat undernutrition within this age group. This includes assessing cost-benefit and potential risks, including assessing whether abruptly increasing nutritional intake among undernourished adolescents may precipitate earlier puberty with consequent shorter stature,^57^ or may alter risks of non-communicable diseases in adulthood. Assessment of the predictive value of preconception and post-conception MUAC for adverse outcomes of pregnancy among adolescent girls, and the efficacy of interventions to mitigate risk in this group would also be valuable.^58^ ^59^ ^60^ ^61^

### Conclusions

Our results confirm that among school age children and adolescents, a new growth reference for MUAC-for-age can be used alongside WHO growth standards and is a valid anthropometric marker of the risk of mortality in HIV infected and uninfected populations in Africa. With its practical simplicity and availability of reference curves, MUAC can be used in place of body mass index to assess communities and guide treatment for individuals at nutrition and HIV programmes, and as a standardised means of assessment in research.

**What is already known on this topic**

School age children and adolescents are vulnerable to malnutrition in many countries, through poverty, famine, and conflict
Nutritional status in this age group is currently assessed by body mass index for age
Although MUAC is the cornerstone of assessment in other age groups, no internationally accepted reference for MUAC exists for school age children and adolescents

**What this study adds**

A new MUAC for age growth reference that accords with WHO standards is an effective marker of the risk of mortality for school age children and adolescents
MUAC can be used to assess communities, to guide treatment for individuals, and as a standardised means of nutritional assessment in research for this age group

## Web extra

Extra material supplied by authors

**Supplementary information:** appendix tables 1-8 and figures 1-9

**Supplementary figure:** MUAC-for-age chart in girls for clinical and programmatic use

**Supplementary figure:** MUAC-for-age chart in boys for clinical and programmatic use

## Notes

We thank the National Center for Health Statistics, National Health and Nutrition Examination Survey Data, Hyattsville, MD: US Department of Health and Human Services, Centers for Disease Control and Prevention for collecting and making publically available the HES/NHANES datasets and those who were examined in order to provide these data; the participants and clinical and research teams of the ARROW trial and Kilifi County Hospital for care provided, data collection and making the data available for this analysis; Mercedes de Onis, at the Growth Assessment and Surveillance Unit, Department of Nutrition, WHO for providing summary MUAC reference data for 60 to 71 month olds; and *The BMJ* reviewers, Saskia van der Kam, Dheeraj Shah, and Tim Cole, for their comments which led to a simpler modelling approach and an improved transition from WHO growth standards at as 5 years, as well as consideration of our findings in relation to currently used cut offs and the compromises involved in their use in operational settings.

Contributors: JAB conceived the study. JAB, LaM, GF and MN prepared the data to generate growth curves. LaM modelled the growth reference curves. SW and DG supervised the collection of the validation dataset from the ARROW trial. MN, MM, LiM and EB collected and processed the Kenyan validation dataset. MN and JAB performed the validation analysis. LaM and JAB wrote the first draft of the manuscript. All authors reviewed and critically edited the final manuscript and agree to be accountable for all aspects of the work in ensuring that questions related to the accuracy or integrity of any part of the work are appropriately investigated and resolved. JAB had full access to all the data in the study and takes responsibility for the integrity of the data and the accuracy of the data analysis. JAB acts as guarantor of the study.

Ethical approval: The NHES and NHANES surveys underwent internal human subjects review, but institutional review board approval using current standards was not obtained at that time. The ARROW trial was approved by the national regulatory and ethics review bodies in Uganda and Zimbabwe, and in the UK and Baylor University in the USA. The analysis of Kenyan data was approved by the Kenya Medical Research Institute (KEMRI) national ethical review committee.

Data sharing: The datasets used to construct MUAC z scores are freely available at www.cdc.gov/nchs/data_access/ftp_data.htm. The statistical code may be requested from Lazarus Mramba (Lazarus.Mramba@medicine.ufl.edu). Validation data from Kenya may be requested from the KEMRI//Wellcome Trust Research Programme Data Governance Committee (dgc@kemri-wellcome.org) and from the ARROW trial may be requested from Sarah Walker (rmjlasw@ucl.ac.uk).

Transparency: The lead author (JAB) affirms that the manuscript is an honest, accurate, and transparent account of the study being reported; that no important aspects of the study have been omitted; and that any discrepancies from the study as planned have been explained.
