# Supplementary material for: A growth reference for mid upper arm circumference for age among school age children and adolescents, and validation for mortality: growth curve construction and longitudinal cohort study
Source: BMJ. 2017 Aug 3;358:j3423. doi: 10.1136/bmj.j3423 (PMC5541507; doi:10.1136/bmj.j3423)
Supplement: Supplementary file 1 — Supplementary information: appendix tables 1-8 and figures 1-9 [file mral036206.ww1.pdf]

Supplementary file

A Growth Reference for Mid Upper Arm Circumference for Age among School Age Children and Adolescents, with Validation for Mortality in Two Cohorts.

Lazarus Mramba, Moses Ngari, Martha Mwangome, Lilian Muchai, Evasius Bauni, A Sarah Walker, Diana M Gibb, Gregory Fegan, James A Berkley.

Supplementary Tables & Figures

Supplementary Table 1: Deviance, Akaike Information Criteria, and Bayesian Information Criteria values for competing models. 2

Supplementary Table 2: Cross tabulation of MUAC and BMI-for-age Z scores amongst children in the ARROW trial. 2

Supplementary Table 3: Areas under Receiver Operating Characteristic Curves for children in the ARROW trial by age and sex. 3

Supplementary Table 4: Simplified cut off values for MUAC-for-Age. 4

Supplementary Table 5: Exploratory analysis of simplified cut offs for MUAC-for-Age, discriminatory performance for mortality in the ARROW trial. 5

Supplementary Table 6: Cross tabulation of MUAC and BMI-for-age Z scores amongst children admitted to Kilifi County Hospital, Kenya and followed up post-discharge. 5

Supplementary Table 7: Areas under Receiver Operating Characteristic Curves for children among children discharged from Kilifi County Hospital by sex. 6

Supplementary Table 8: Exploratory analysis of simplified cut offs for MUAC-for-Age, discriminatory performance for mortality among children discharged from Kilifi County Hospital. .. 6

Supplementary Figure 1: Merged data and modelled Z Scores. 7

Supplementary Figure 2: Residual plots for BCCG models. 8

Supplementary Figure 4: Worm plots for BCCG models 9

Supplementary Figure 7: A simplified MUAC cut-off compared to -2 & -3Z Scores. 10

Supplementary Figure 8: MUAC values identified by MUACz <-2, a simplified MUAC cut-off, and BMIz <-2 in the ARROW trial. 11

Supplementary Figure 9: MUAC values identified by MUACz <-2, a simplified MUAC cut-off, and BMIz <-2 among children discharged from Kilifi County Hospital. 12

MUAC-for-Age Z Score and Percentile Tables

Z score values for girls and boys........ 13

Percentile values for girls and boys........ 19

Supplementary Table 1: Deviance, Akaike Information Criteria, and Bayesian Information Criteria values for competing models.

	Deviance	AIC (df)	BIC (df)
**Models for Girls** (values displayed minus 120,000)			
BCT	302.3	335.6 (16.7)	474.0 (16.7)
BCPE	196.8	267.6 (35.4)	561.5 (35.4)
BCCG	300.7	334.4 (16.8)	473.9 (16.8)
**Models for Boys** (values displayed minus 114,000)			
BCT	127.6	173.0 (22.7)	361.3 (22.7)
BCPE	112.5	175.6 (31.6)	437.2 (31.6)
BCCG	221.4	265.3 (21.9)	447.2 (21.9)

BCT (Box-Cox t distribution); BCPE (Box-Cox Power Exponential); BCCG (Box-Cox-Cole-Green)

BCT and BCPE transformations model both skewness and kurtosis, while BCCG accounts for skewness only. They typically differ in how outliers are represented. BCCG allows the parameters: L (transformation), M (median) and S (skew) to be interpretable as characteristics of the original data, and thereby used in typical anthropometric software, such as WHO Anthro. This output has also been achieved by using BCT and BPPE with fixed value for kurtosis to eliminate it (effectively the BCCG method), or by generating LMS values derived from the final smoothed curves irrespective of methods used to model the original data.

Supplementary Table 2: Cross tabulation of MUAC and BMI-for-age Z scores amongst children in the ARROW trial.

**All children**		**BMI-for-age Z score**			
		**-2 or more**	**-3 to -2**	**<-3**	**Total**
	**-2 or more**	455	9	3	467
	**-3 to -2**	95	20	3	118
**MUAC for age Z score**	**<-3**	33	29	38	100
	**Total**	583	58	44	685

**Children that died**		**BMI-for-age Z score**			
		**-2 or more**	**-3 to -2**	**<-3**	**Total**
	**-2 or more**	4	0	0	4
	**-3 to -2**	3	0	1	4
**MUAC for age Z score**	**<-3**	2	2	6	10
	**Total**	9	2	7	18

Supplementary Table 3: Areas under Receiver Operating Characteristic Curves for children in the ARROW trial by age and sex.

	5 to 9 year olds			10 to 17 year olds		
	**MUACz**	**BMIz**	**P****	**MUACz**	**BMIz**	**P****
**n**	436	436		249	249	
**AUC (95% CI)***	0.75 (0.54 to 0.95)	0.61 (0.41 to 0.80)	0.3	0.85 (0.74 to 0.96)	0.80 (0.66 to 0.94)	0.4

	Boys			Girls		
	**MUACz**	**BMIz**	**P****	**MUACz**	**BMIz**	**P****
**n**	436	436		249	249	
**AUC (95% CI)***	0.76 (0.59 to 0.92)	0.69 (0.53 to 0.85)	0.4	0.87 † (0.75 to 0.99)	0.84 †† (0.68 to 0.99)	0.6

	Boys			Girls		
	**5 to 9y**	**10 to 17y**	**P§**	**5 to 9y**	**10 to 17y**	**P§**
	**MUACz**	**MUACz**		**MUACz**	**MUACz**	
**n**	224	117		211	133	
**AUC (95% CI)***	0.71 (0.45 to 0.97)	0.81 (0.60 to 0.99)	0.6	0.85 (0.58 to 0.99)	0.89 (0.74 to 0.99)	0.5

* AUC: area under the receiver operating characteristic curve
** P values for MUACz vs. BMIz
† MUACz (boys) vs MUACz (girls) P=0.2
†† BMIz (boys) vs BMIz (girls) P=0.2
§ P values 5 to 9 year olds vs. 10 to 17 year olds

Supplementary Table 4: Simplified cut off values for MUAC-for-Age.

Age in years	Simplified MUAC cut off
(6m to 4y)	(12.5)
5	13.1
6	13.6
7	14.2
8	14.8
9	15.3
10	15.9
11	16.5
12	17.0
13	17.6
14	18.2
15	18.7
16	19.3
17	19.9
18	20.4
19	21.0

Simplified cut off values are assigned according to age in completed years in a linear slope from 12.5cm at <5 years to 21cm at 19 years, which are commonly used fixed MUAC cut offs for young children and adults. The formula is:

$$\boldsymbol{cutoff = 12.5 + \left(\frac{17}{30} * (age\ in\ years - 4)\right)}$$

Supplementary Table 5: Exploratory analysis of simplified cut offs for MUAC-for-Age, discriminatory performance for mortality in the ARROW trial.

	MUAC <SCO* N=685	MUACz continuous N=685	MUACz <-3 N=685	MUACz <-2 N=685	BMIz <-2 N=685
**Number identified (%, caseload)**	164 (24)	-	100 (15)	218 (32)	102 (15)
**Deaths identified (%, sensitivity)**	13 (72)	-	10 (56)	14 (7)	9 (50)
**Hazard ratio for death (95% CI)****	9.1 (2.86 – 23.0)	-	11.1 (3.40 – 36.0)	3.63 (0.90 – 14.7)	1.91 (0.41 – 8.91)
**AUC (95% CI)**†	0.75 (0.64 to 0.86)	0.81 (0.70 to 0.92)	0.71 (0.59 – 0.83)	0.74 (0.64 to 0.84)	0.68 (0.56 to 0.80)
**P value**§	-	0.1	0.4	0.8	0.2

* SCO: Simplified Cut Off
** Adjusted for age and sex
† AUC: area under the receiver operating characteristic curve
§ P value for AUCs compared to Simplified Cut Off

Supplementary Table 6: Cross tabulation of MUAC and BMI-for-age Z scores amongst children admitted to Kilifi County Hospital, Kenya and followed up post-discharge.

**All children**		**BMI-for-age Z score**				
		**-2 or more**	**-3 to -2**	**<-3**	**Missing**	**Total**
	**-2 or more**	881	81	43	28	1033
	**-3 to -2**	270	114	33	8	425
**MUAC for age Z score**	**<-3**	97	82	90	6	275
	**Missing**	8	0	0	0	8
	**Total**	1,256	277	166	42	1,741

**Children that died**		**BMI-for-age Z score**				
		**-2 or more**	**-3 to -2**	**<-3**	**Missing**	**Total**
	**-2 or more**	12	1	0	0	13
	**-3 to -2**	9	2	2	0	13
**MUAC for age Z score**	**<-3**	7	3	10	0	20
	**Missing**	0	0	0	0	0
	**Total**	28	6	12	0	46

Supplementary Table 7: Areas under Receiver Operating Characteristic Curves for children among children discharged from Kilifi County Hospital by sex.

	Boys aged 5 to 13 years			Girls aged 5 to 13 years		
	**MUACz**	**BMIz**	**P****	**MUACz**	**BMIz**	**P****
**n**	944	436		736	249	
**AUC (95% CI)***	0.68 (0.57 to 0.78)	0.52 (0.39 to 0.66)	0.01	0.80 † (0.71 to 0.89)	0.64 § (0.53 to 0.76)	0.003

* AUC: area under the receiver operating characteristic curve
** P value for MUACz vs. BMIz AUCs
† MUACz (all boys) vs MUACz (all girls) P=0.09
§ BMIz (all boys) vs BMIz (all girls) P=0.2

Supplementary Table 8: Exploratory analysis of simplified cut offs for MUAC-for-Age, discriminatory performance for mortality among children discharged from Kilifi County Hospital.

	**MUAC <SCO* N=1,680**	**MUACz continuous N=1,680**	**MUACz <-3 N=1,680**	**MUACz <-2 N=1,680**	**BMIz <-2 N=1,633**
**Number identified (%, caseload)**	488 (29)	-	275 (16)	700 (40)	435 (26)
**Deaths identified (%, sensitivity)**	23 (50)	-	20 (43)	33 (72)	18 (39)
**Hazard ratio for death (95% CI)****	2.25 (1.23 – 4.1)	-	3.74 (2.02 to 6.9)	3.35 (1.74 to 6.5`)	1.78 (0.97 – 3.27)
**AUC (95% CI)** †	0.61 (0.53 to 0.68)	0.73 (0.65 to 0.80)	0.64 (0.57 – 0.72)	0.66 (0.59 to 0.73)	0.57 (0.50 to 0.64)
**P value**§	-	<0.0001	0.09	0.06	0.2

* SCO: Simplified Cut Off
** Adjusted for age, sex and HIV status
† AUC: area under the receiver operating characteristic curve
§ P value for AUC compared to Simplified Cut Off

Supplementary Figure 1: Merged data and modelled Z Scores.

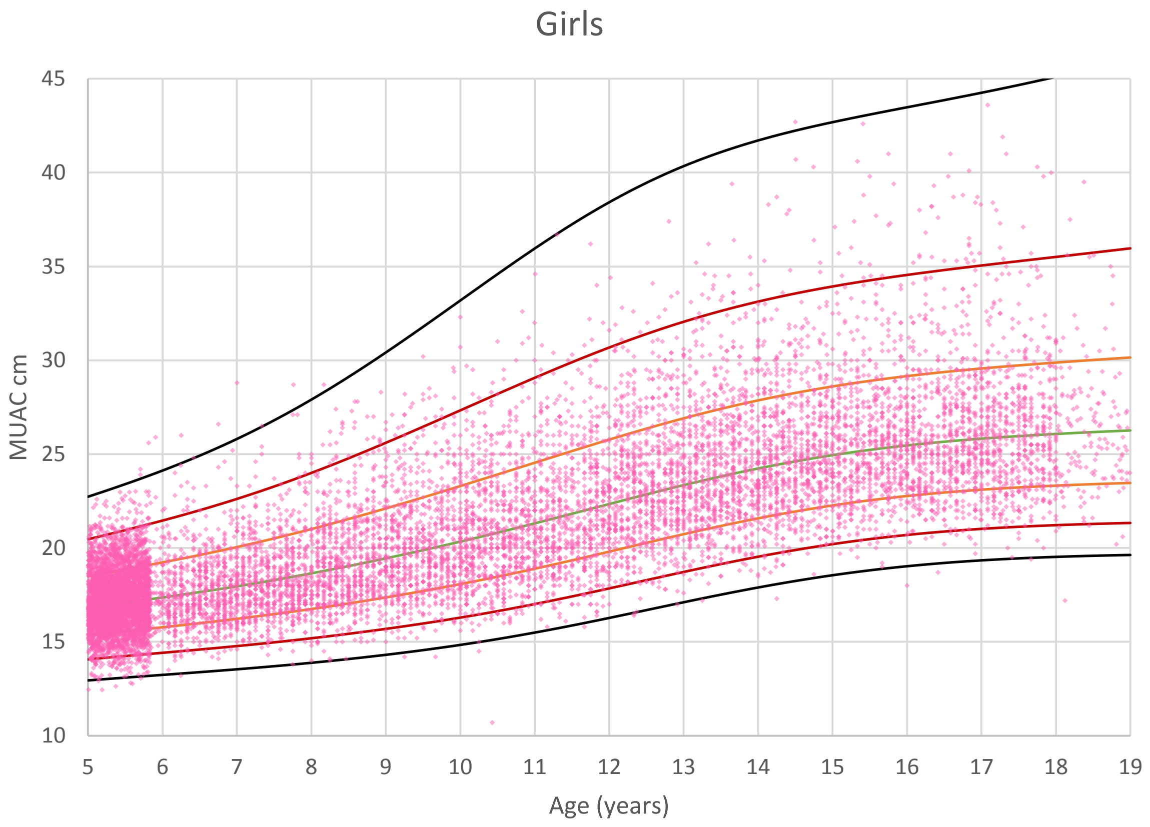

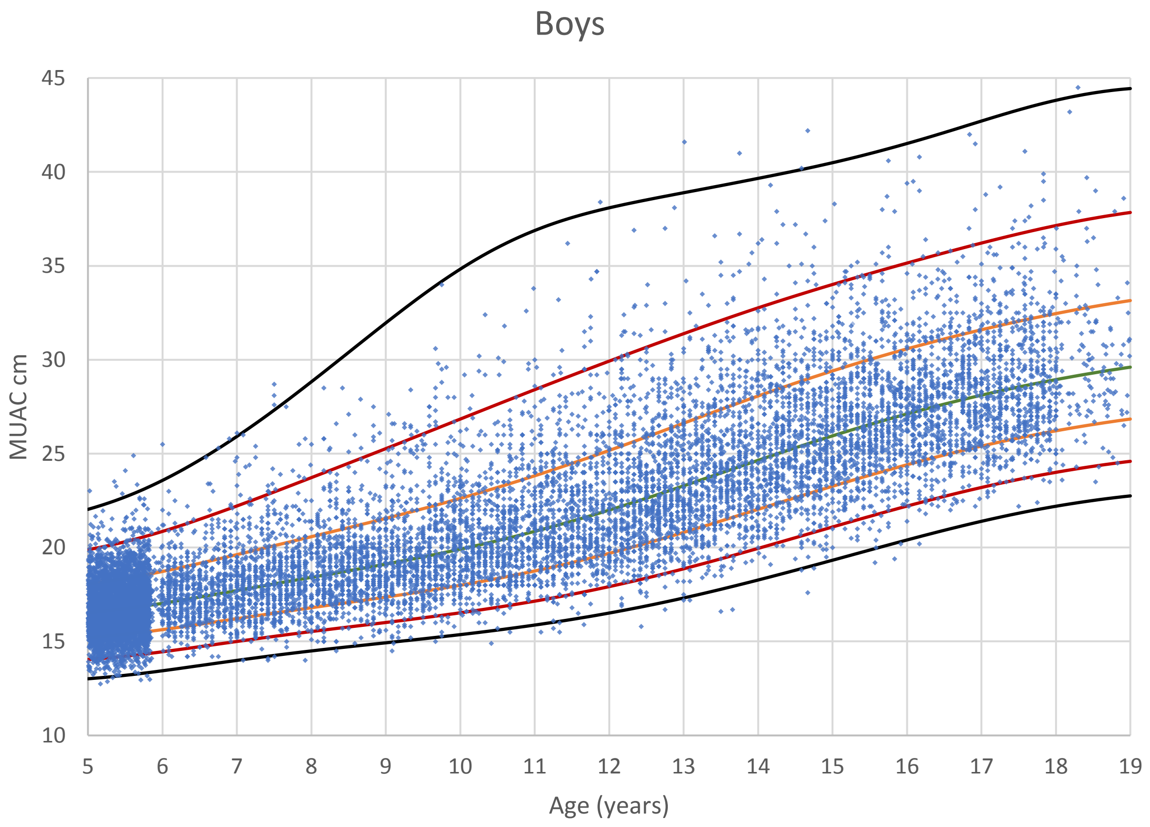

Supplementary Figure 2: Residual plots for BCCG models.

Girls

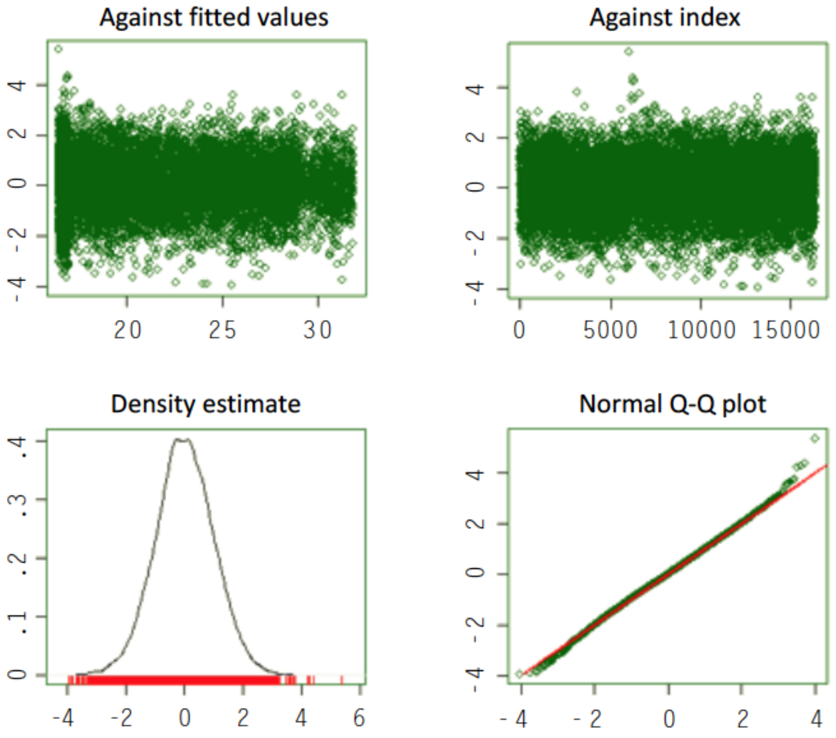

Boys

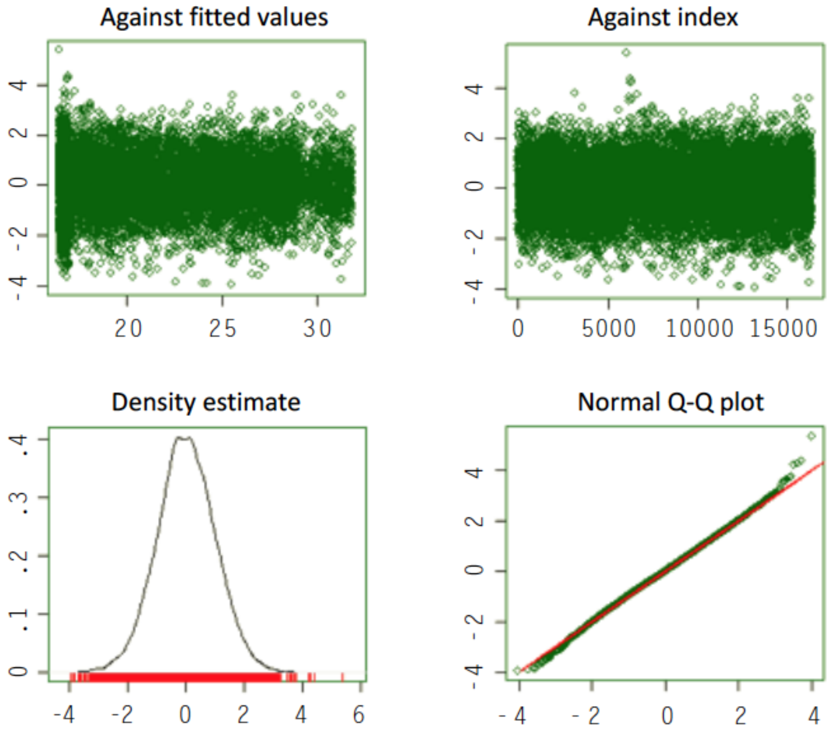

Supplementary Figure 4: Worm plots for BCCG models

Girls

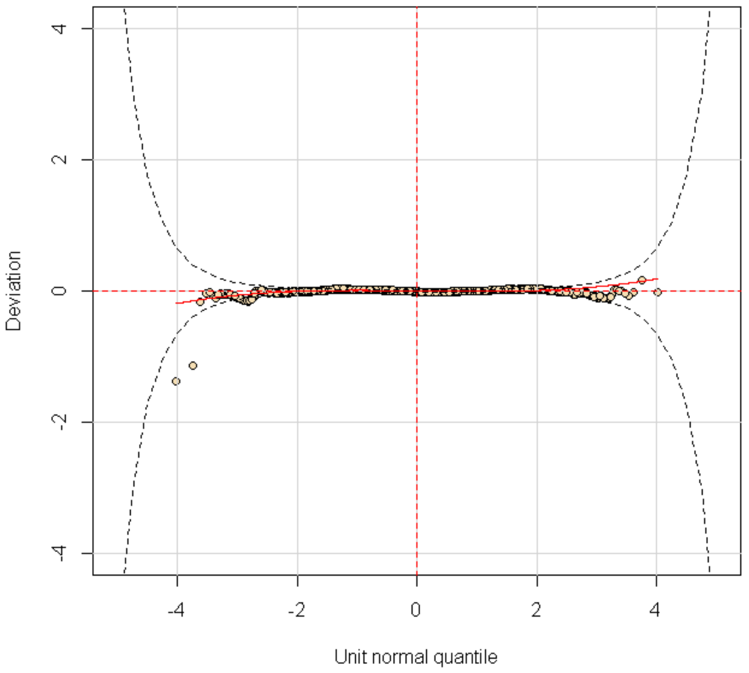

Boys

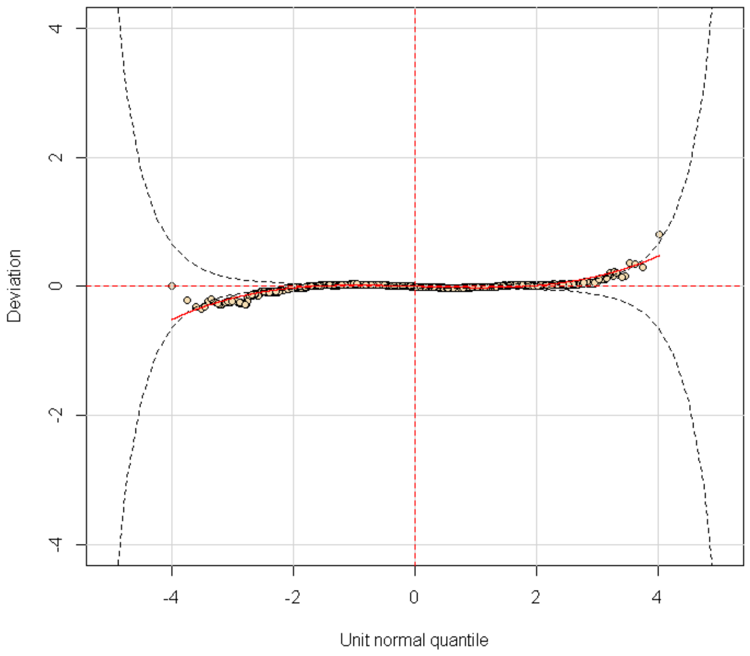

Supplementary Figure 7: A simplified MUAC cut-off compared to -2 & -3Z Scores.

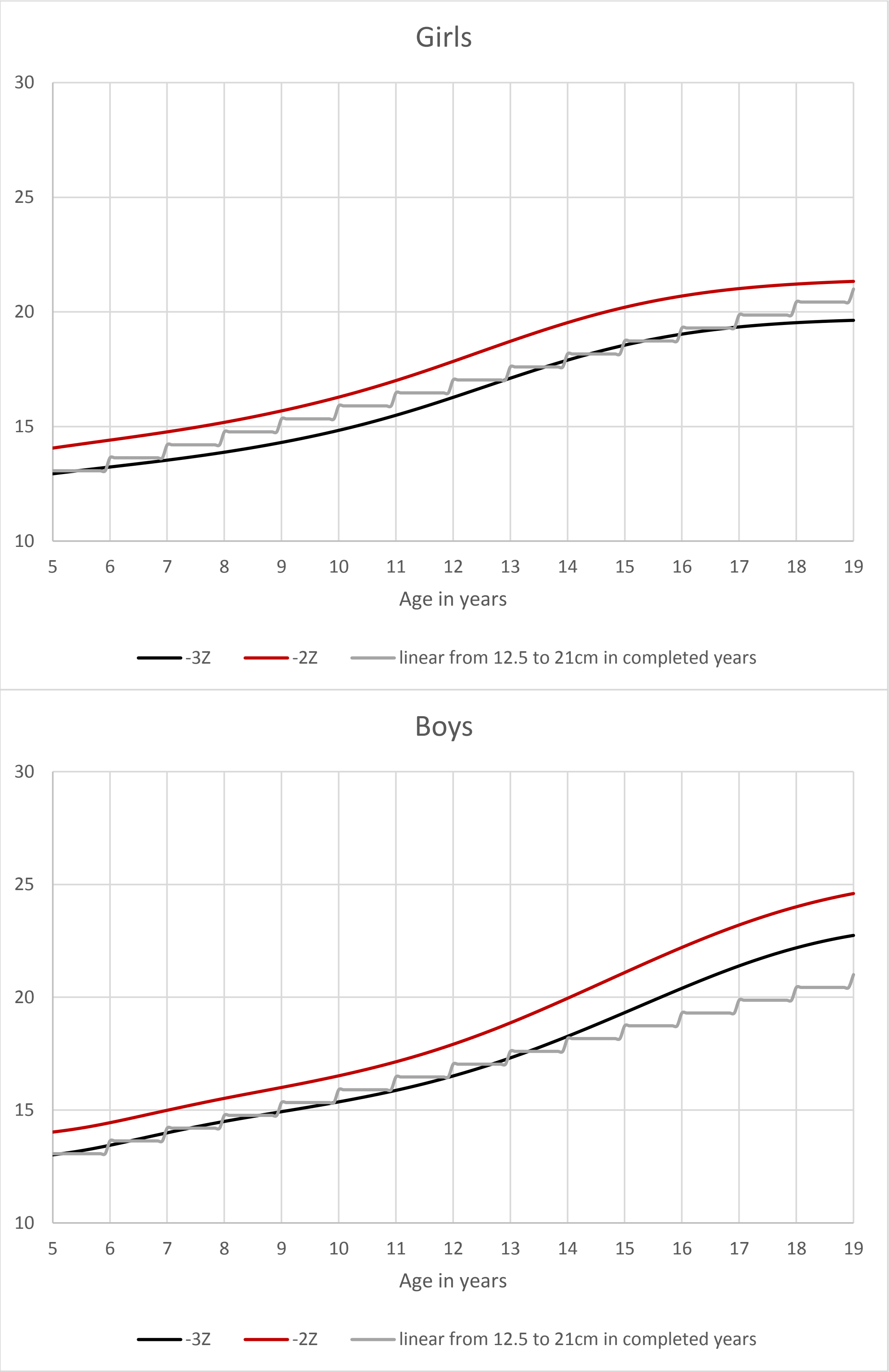

Supplementary Figure 8: MUAC values identified by MUACz <-2, a simplified MUAC cut-off, and BMIz <-2 in the ARROW trial.

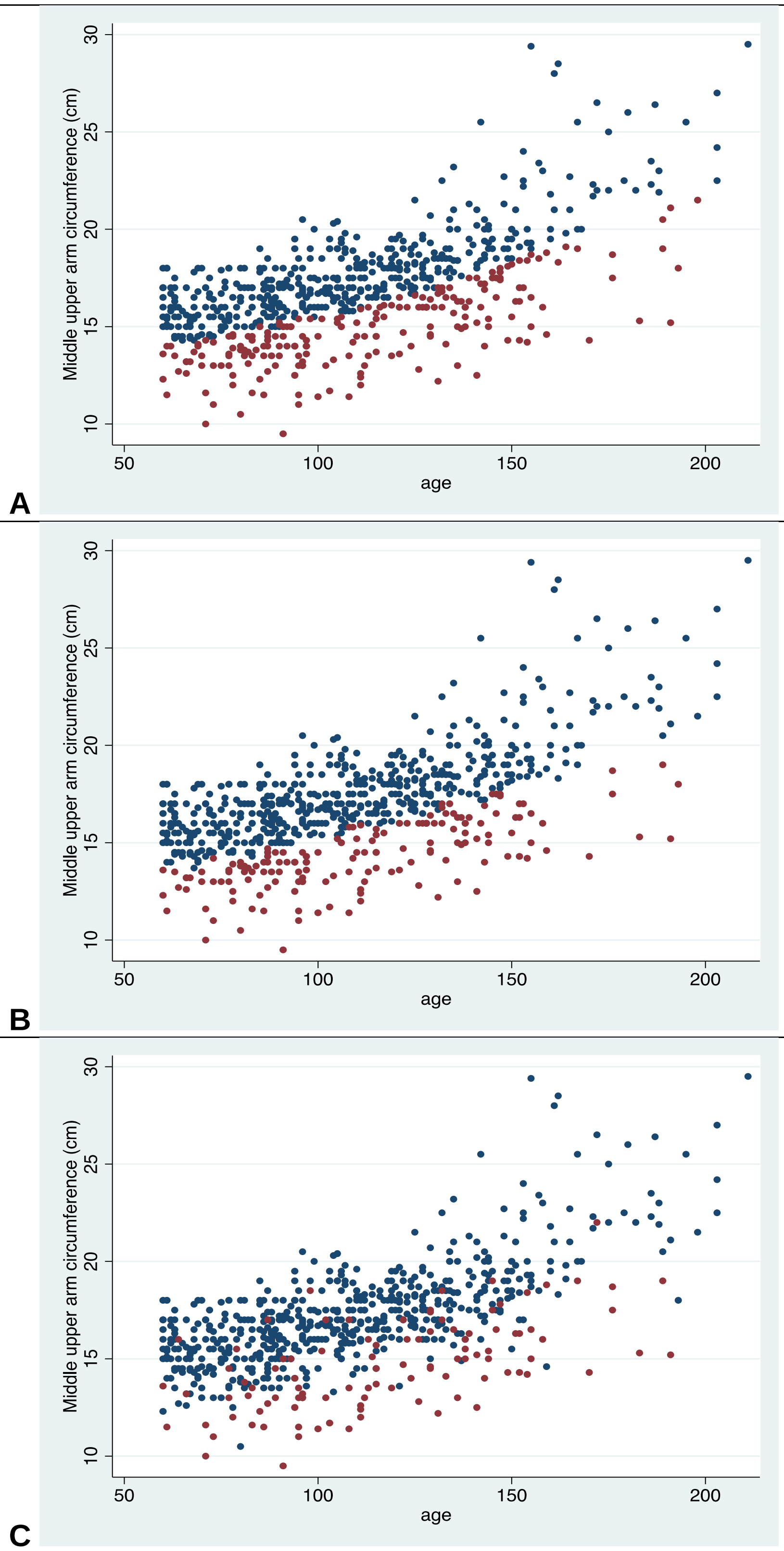

MUAC by age in months among ARROW Trial Participants, above and below cut offs of A: MUACz <-2; B Simplified MUAC < linear slope from 12.5 to 21cm; C: BMIz <-2. Blue = above cut off; Red = below cut off.

Supplementary Figure 9: MUAC values identified by MUACz <-2, a simplified MUAC cut-off, and BMIz <-2 among children discharged from Kilifi County Hospital.

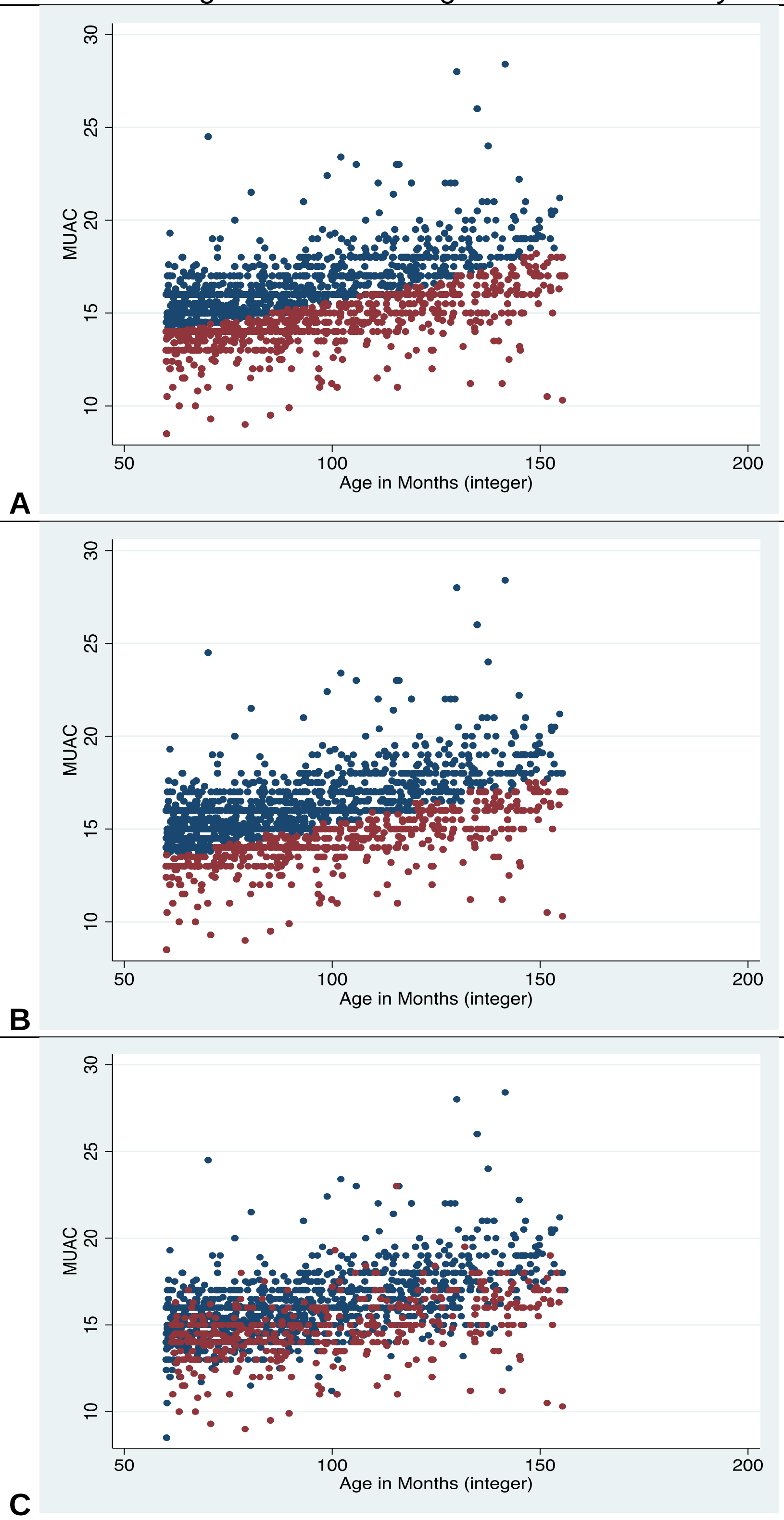

MUAC by age in months among ARROW Trial Participants, above and below cut offs of A: MUACz <-2; B Simplified MUAC < linear slope from 12.5 to 21cm; C: BMIz <-2. Blue = above cut off; Red = below cut off.

Mid-upper arm circumference-for-age BOYS (5 to 19 years)

								Z Scores (MUAC in cm)				
Year:Month	Month	L	M	S	-3	-2	-1	0	+1	+2	+3
5 : 0	60	-0.72895	16.51891	0.08670	13.0	14.0	15.2	16.5	18.1	19.9	22.0
5 : 1	61	-0.75845	16.55158	0.08699	13.0	14.1	15.2	16.6	18.1	19.9	22.1
5 : 2	62	-0.78927	16.58562	0.08728	13.1	14.1	15.2	16.6	18.2	20.0	22.2
5 : 3	63	-0.82138	16.62121	0.08758	13.1	14.1	15.3	16.6	18.2	20.1	22.3
5 : 4	64	-0.85473	16.65854	0.08789	13.1	14.1	15.3	16.7	18.3	20.2	22.5
5 : 5	65	-0.88928	16.69781	0.08819	13.2	14.2	15.3	16.7	18.3	20.2	22.6
5 : 6	66	-0.92496	16.73915	0.08851	13.2	14.2	15.4	16.7	18.4	20.3	22.7
5 : 7	67	-0.96164	16.78248	0.08882	13.2	14.2	15.4	16.8	18.4	20.4	22.8
5 : 8	68	-0.99919	16.82768	0.08915	13.3	14.3	15.5	16.8	18.5	20.5	23.0
5 : 9	69	-1.03745	16.87463	0.08948	13.3	14.3	15.5	16.9	18.5	20.6	23.1
5 : 10	70	-1.07629	16.92319	0.08981	13.4	14.4	15.5	16.9	18.6	20.7	23.3
5 : 11	71	-1.11556	16.97324	0.09015	13.4	14.4	15.6	17.0	18.7	20.8	23.4
6 : 0	72	-1.15511	17.02466	0.09049	13.4	14.4	15.6	17.0	18.7	20.9	23.6
6 : 1	73	-1.19480	17.07731	0.09084	13.5	14.5	15.7	17.1	18.8	21.0	23.7
6 : 2	74	-1.23449	17.13106	0.09120	13.5	14.5	15.7	17.1	18.9	21.1	23.9
6 : 3	75	-1.27403	17.18581	0.09156	13.6	14.6	15.8	17.2	18.9	21.2	24.1
6 : 4	76	-1.31328	17.24140	0.09193	13.6	14.6	15.8	17.2	19.0	21.3	24.3
6 : 5	77	-1.35210	17.29773	0.09231	13.7	14.7	15.9	17.3	19.1	21.4	24.5
6 : 6	78	-1.39034	17.35466	0.09269	13.7	14.7	15.9	17.4	19.2	21.5	24.7
6 : 7	79	-1.42785	17.41207	0.09308	13.8	14.8	16.0	17.4	19.2	21.6	24.9
6 : 8	80	-1.46450	17.46982	0.09347	13.8	14.8	16.0	17.5	19.3	21.7	25.1
6 : 9	81	-1.50018	17.52784	0.09388	13.9	14.9	16.1	17.5	19.4	21.9	25.3
6 : 10	82	-1.53485	17.58607	0.09428	13.9	14.9	16.1	17.6	19.5	22.0	25.5
6 : 11	83	-1.56850	17.64449	0.09470	13.9	14.9	16.2	17.6	19.5	22.1	25.7
7 : 0	84	-1.60108	17.70307	0.09512	14.0	15.0	16.2	17.7	19.6	22.2	25.9
7 : 1	85	-1.63258	17.76178	0.09555	14.0	15.0	16.3	17.8	19.7	22.3	26.1
7 : 2	86	-1.66297	17.82059	0.09598	14.1	15.1	16.3	17.8	19.8	22.5	26.4
7 : 3	87	-1.69222	17.87946	0.09642	14.1	15.1	16.4	17.9	19.9	22.6	26.6
7 : 4	88	-1.72030	17.93836	0.09687	14.2	15.2	16.4	17.9	19.9	22.7	26.8
7 : 5	89	-1.74719	17.99727	0.09732	14.2	15.2	16.5	18.0	20.0	22.8	27.1
7 : 6	90	-1.77285	18.05615	0.09778	14.3	15.3	16.5	18.1	20.1	23.0	27.3
7 : 7	91	-1.79727	18.11498	0.09824	14.3	15.3	16.5	18.1	20.2	23.1	27.6
7 : 8	92	-1.82040	18.17371	0.09871	14.3	15.4	16.6	18.2	20.3	23.2	27.8
7 : 9	93	-1.84224	18.23233	0.09918	14.4	15.4	16.6	18.2	20.3	23.3	28.1
7 : 10	94	-1.86274	18.29080	0.09966	14.4	15.4	16.7	18.3	20.4	23.5	28.3
7 : 11	95	-1.88189	18.34908	0.10014	14.5	15.5	16.7	18.3	20.5	23.6	28.6
8 : 0	96	-1.89968	18.40721	0.10063	14.5	15.5	16.8	18.4	20.6	23.7	28.8
8 : 1	97	-1.91614	18.46524	0.10112	14.5	15.6	16.8	18.5	20.7	23.8	29.1
8 : 2	98	-1.93128	18.52324	0.10162	14.6	15.6	16.9	18.5	20.7	24.0	29.3
8 : 3	99	-1.94514	18.58127	0.10211	14.6	15.6	16.9	18.6	20.8	24.1	29.6
8 : 4	100	-1.95773	18.63939	0.10262	14.6	15.7	17.0	18.6	20.9	24.2	29.9
8 : 5	101	-1.96908	18.69765	0.10312	14.7	15.7	17.0	18.7	21.0	24.4	30.1
8 : 6	102	-1.97920	18.75613	0.10363	14.7	15.8	17.1	18.8	21.1	24.5	30.4
8 : 7	103	-1.98813	18.81489	0.10414	14.8	15.8	17.1	18.8	21.1	24.6	30.7
8 : 8	104	-1.99588	18.87398	0.10465	14.8	15.8	17.2	18.9	21.2	24.7	30.9
8 : 9	105	-2.00248	18.93347	0.10516	14.8	15.9	17.2	18.9	21.3	24.9	31.2
8 : 10	106	-2.00795	18.99342	0.10568	14.9	15.9	17.3	19.0	21.4	25.0	31.4
8 : 11	107	-2.01231	19.05389	0.10619	14.9	16.0	17.3	19.1	21.5	25.1	31.7
9 : 0	108	-2.01558	19.11495	0.10671	14.9	16.0	17.4	19.1	21.6	25.3	32.0
9 : 1	109	-2.01780	19.17665	0.10722	15.0	16.0	17.4	19.2	21.6	25.4	32.2
9 : 2	110	-2.01897	19.23906	0.10774	15.0	16.1	17.5	19.2	21.7	25.5	32.5
9 : 3	111	-2.01912	19.30221	0.10825	15.0	16.1	17.5	19.3	21.8	25.7	32.7
9 : 4	112	-2.01828	19.36613	0.10877	15.1	16.2	17.6	19.4	21.9	25.8	33.0
9 : 5	113	-2.01647	19.43088	0.10928	15.1	16.2	17.6	19.4	22.0	25.9	33.2
9 : 6	114	-2.01371	19.49646	0.10979	15.1	16.3	17.7	19.5	22.1	26.1	33.5
9 : 7	115	-2.01004	19.56293	0.11029	15.2	16.3	17.7	19.6	22.2	26.2	33.7
9 : 8	116	-2.00546	19.63031	0.11080	15.2	16.3	17.8	19.6	22.3	26.3	33.9
9 : 9	117	-2.00000	19.69863	0.11130	15.3	16.4	17.8	19.7	22.3	26.4	34.2

Year:Month			Month	L	M	S	Z Scores (MUAC in cm) -3	-2	-1	0	+1	+2	+3
9	:	10	118	-1.99370	19.76794	0.11179	15.3	16.4	17.9	19.8	22.4	26.6	34.4
9	:	11	119	-1.98657	19.83825	0.11228	15.3	16.5	17.9	19.8	22.5	26.7	34.6
10	:	0	120	-1.97863	19.90962	0.11277	15.4	16.5	18.0	19.9	22.6	26.8	34.8
10	:	1	121	-1.96991	19.98207	0.11325	15.4	16.6	18.0	20.0	22.7	27.0	35.0
10	:	2	122	-1.96043	20.05563	0.11372	15.4	16.6	18.1	20.1	22.8	27.1	35.2
10	:	3	123	-1.95022	20.13034	0.11419	15.5	16.7	18.2	20.1	22.9	27.2	35.4
10	:	4	124	-1.93930	20.20623	0.11465	15.5	16.7	18.2	20.2	23.0	27.4	35.6
10	:	5	125	-1.92770	20.28334	0.11510	15.6	16.8	18.3	20.3	23.1	27.5	35.8
10	:	6	126	-1.91544	20.36166	0.11554	15.6	16.8	18.3	20.4	23.2	27.6	36.0
10	:	7	127	-1.90255	20.44122	0.11597	15.7	16.9	18.4	20.4	23.3	27.8	36.1
10	:	8	128	-1.88906	20.52201	0.11640	15.7	16.9	18.5	20.5	23.4	27.9	36.3
10	:	9	129	-1.87501	20.60406	0.11681	15.7	17.0	18.5	20.6	23.5	28.0	36.5
10	:	10	130	-1.86041	20.68738	0.11721	15.8	17.0	18.6	20.7	23.6	28.1	36.6
10	:	11	131	-1.84530	20.77196	0.11761	15.8	17.1	18.7	20.8	23.7	28.3	36.7
11	:	0	132	-1.82971	20.85783	0.11798	15.9	17.1	18.7	20.9	23.8	28.4	36.9
11	:	1	133	-1.81367	20.94498	0.11835	15.9	17.2	18.8	20.9	23.9	28.5	37.0
11	:	2	134	-1.79720	21.03344	0.11870	16.0	17.3	18.9	21.0	24.0	28.7	37.1
11	:	3	135	-1.78034	21.12321	0.11904	16.0	17.3	19.0	21.1	24.1	28.8	37.2
11	:	4	136	-1.76312	21.21431	0.11937	16.1	17.4	19.0	21.2	24.3	28.9	37.4
11	:	5	137	-1.74555	21.30673	0.11967	16.1	17.4	19.1	21.3	24.4	29.0	37.5
11	:	6	138	-1.72768	21.40050	0.11997	16.2	17.5	19.2	21.4	24.5	29.2	37.6
11	:	7	139	-1.70954	21.49562	0.12024	16.2	17.6	19.3	21.5	24.6	29.3	37.7
11	:	8	140	-1.69114	21.59208	0.12050	16.3	17.6	19.3	21.6	24.7	29.4	37.8
11	:	9	141	-1.67254	21.68983	0.12075	16.3	17.7	19.4	21.7	24.8	29.6	37.8
11	:	10	142	-1.65377	21.78883	0.12097	16.4	17.8	19.5	21.8	24.9	29.7	37.9
11	:	11	143	-1.63486	21.88905	0.12118	16.5	17.8	19.6	21.9	25.1	29.8	38.0
12	:	0	144	-1.61586	21.99043	0.12137	16.5	17.9	19.7	22.0	25.2	29.9	38.1
12	:	1	145	-1.59680	22.09294	0.12154	16.6	18.0	19.8	22.1	25.3	30.1	38.2
12	:	2	146	-1.57771	22.19654	0.12169	16.6	18.1	19.9	22.2	25.4	30.2	38.2
12	:	3	147	-1.55864	22.30118	0.12183	16.7	18.1	19.9	22.3	25.5	30.3	38.3
12	:	4	148	-1.53962	22.40682	0.12194	16.8	18.2	20.0	22.4	25.6	30.4	38.4
12	:	5	149	-1.52070	22.51341	0.12204	16.8	18.3	20.1	22.5	25.8	30.5	38.4
12	:	6	150	-1.50190	22.62092	0.12212	16.9	18.4	20.2	22.6	25.9	30.7	38.5
12	:	7	151	-1.48326	22.72931	0.12218	17.0	18.5	20.3	22.7	26.0	30.8	38.6
12	:	8	152	-1.46482	22.83852	0.12221	17.0	18.5	20.4	22.8	26.1	30.9	38.6
12	:	9	153	-1.44663	22.94852	0.12223	17.1	18.6	20.5	22.9	26.3	31.0	38.7
12	:	10	154	-1.42870	23.05927	0.12223	17.2	18.7	20.6	23.1	26.4	31.1	38.8
12	:	11	155	-1.41108	23.17069	0.12221	17.2	18.8	20.7	23.2	26.5	31.3	38.8
13	:	0	156	-1.39378	23.28271	0.12217	17.3	18.9	20.8	23.3	26.6	31.4	38.9
13	:	1	157	-1.37683	23.39523	0.12211	17.4	19.0	20.9	23.4	26.7	31.5	39.0
13	:	2	158	-1.36024	23.50818	0.12204	17.5	19.0	21.0	23.5	26.9	31.6	39.0
13	:	3	159	-1.34404	23.62148	0.12194	17.5	19.1	21.1	23.6	27.0	31.7	39.1
13	:	4	160	-1.32825	23.73504	0.12183	17.6	19.2	21.2	23.7	27.1	31.9	39.1
13	:	5	161	-1.31289	23.84879	0.12170	17.7	19.3	21.3	23.8	27.2	32.0	39.2
13	:	6	162	-1.29798	23.96263	0.12155	17.8	19.4	21.4	24.0	27.4	32.1	39.3
13	:	7	163	-1.28355	24.07649	0.12138	17.9	19.5	21.5	24.1	27.5	32.2	39.3
13	:	8	164	-1.26961	24.19029	0.12120	17.9	19.6	21.6	24.2	27.6	32.3	39.4
13	:	9	165	-1.25619	24.30394	0.12101	18.0	19.7	21.7	24.3	27.7	32.4	39.5
13	:	10	166	-1.24331	24.41736	0.12079	18.1	19.8	21.8	24.4	27.8	32.5	39.5
13	:	11	167	-1.23099	24.53048	0.12057	18.2	19.9	21.9	24.5	28.0	32.7	39.6
14	:	0	168	-1.21926	24.64320	0.12032	18.3	20.0	22.0	24.6	28.1	32.8	39.7
14	:	1	169	-1.20812	24.75546	0.12007	18.4	20.0	22.1	24.8	28.2	32.9	39.7
14	:	2	170	-1.19761	24.86720	0.11980	18.4	20.1	22.2	24.9	28.3	33.0	39.8
14	:	3	171	-1.18774	24.97838	0.11952	18.5	20.2	22.3	25.0	28.4	33.1	39.9
14	:	4	172	-1.17852	25.08897	0.11922	18.6	20.3	22.4	25.1	28.5	33.2	39.9
14	:	5	173	-1.16998	25.19891	0.11892	18.7	20.4	22.5	25.2	28.6	33.3	40.0
14	:	6	174	-1.16213	25.30817	0.11860	18.8	20.5	22.6	25.3	28.8	33.4	40.1
14	:	7	175	-1.15499	25.41670	0.11828	18.9	20.6	22.7	25.4	28.9	33.5	40.1
14	:	8	176	-1.14858	25.52447	0.11795	19.0	20.7	22.9	25.5	29.0	33.6	40.2

Year:Month			Month	L	M	S	-3	-2	-1	0	+1	+2	+3
							Z Scores (MUAC in cm)						
14	:	9	177	-1.14291	25.63143	0.11761	19.1	20.8	23.0	25.6	29.1	33.7	40.3
14	:	10	178	-1.13800	25.73753	0.11726	19.1	20.9	23.1	25.7	29.2	33.8	40.3
14	:	11	179	-1.13388	25.84274	0.11691	19.2	21.0	23.2	25.8	29.3	33.9	40.4
15	:	0	180	-1.13055	25.94701	0.11655	19.3	21.1	23.3	25.9	29.4	34.0	40.5
15	:	1	181	-1.12804	26.05030	0.11619	19.4	21.2	23.4	26.1	29.5	34.1	40.6
15	:	2	182	-1.12636	26.15257	0.11583	19.5	21.3	23.5	26.2	29.6	34.2	40.6
15	:	3	183	-1.12553	26.25377	0.11546	19.6	21.4	23.6	26.3	29.7	34.3	40.7
15	:	4	184	-1.12555	26.35388	0.11510	19.7	21.5	23.7	26.4	29.8	34.4	40.8
15	:	5	185	-1.12638	26.45289	0.11473	19.8	21.6	23.7	26.5	29.9	34.5	40.9
15	:	6	186	-1.12800	26.55079	0.11436	19.9	21.7	23.8	26.6	30.0	34.6	41.0
15	:	7	187	-1.13037	26.64757	0.11399	20.0	21.8	23.9	26.6	30.1	34.7	41.1
15	:	8	188	-1.13345	26.74322	0.11362	20.0	21.8	24.0	26.7	30.2	34.8	41.1
15	:	9	189	-1.13722	26.83773	0.11326	20.1	21.9	24.1	26.8	30.3	34.9	41.2
15	:	10	190	-1.14163	26.93110	0.11290	20.2	22.0	24.2	26.9	30.4	35.0	41.3
15	:	11	191	-1.14666	27.02332	0.11254	20.3	22.1	24.3	27.0	30.5	35.1	41.4
16	:	0	192	-1.15227	27.11438	0.11219	20.4	22.2	24.4	27.1	30.6	35.2	41.5
16	:	1	193	-1.15843	27.20427	0.11184	20.5	22.3	24.5	27.2	30.7	35.2	41.6
16	:	2	194	-1.16511	27.29299	0.11150	20.6	22.4	24.6	27.3	30.8	35.3	41.7
16	:	3	195	-1.17226	27.38052	0.11116	20.7	22.5	24.7	27.4	30.8	35.4	41.8
16	:	4	196	-1.17986	27.46686	0.11083	20.7	22.6	24.7	27.5	30.9	35.5	41.9
16	:	5	197	-1.18788	27.55200	0.11051	20.8	22.6	24.8	27.6	31.0	35.6	42.0
16	:	6	198	-1.19627	27.63593	0.11020	20.9	22.7	24.9	27.6	31.1	35.7	42.1
16	:	7	199	-1.20499	27.71865	0.10990	21.0	22.8	25.0	27.7	31.2	35.8	42.2
16	:	8	200	-1.21395	27.80016	0.10960	21.1	22.9	25.1	27.8	31.3	35.9	42.3
16	:	9	201	-1.22310	27.88048	0.10932	21.2	23.0	25.2	27.9	31.4	36.0	42.4
16	:	10	202	-1.23235	27.95959	0.10904	21.2	23.0	25.2	28.0	31.4	36.0	42.5
16	:	11	203	-1.24164	28.03751	0.10878	21.3	23.1	25.3	28.0	31.5	36.1	42.6
17	:	0	204	-1.25090	28.11425	0.10852	21.4	23.2	25.4	28.1	31.6	36.2	42.7
17	:	1	205	-1.26004	28.18980	0.10827	21.5	23.3	25.5	28.2	31.7	36.3	42.8
17	:	2	206	-1.26901	28.26416	0.10803	21.5	23.4	25.5	28.3	31.7	36.4	42.9
17	:	3	207	-1.27772	28.33735	0.10780	21.6	23.4	25.6	28.3	31.8	36.5	43.0
17	:	4	208	-1.28612	28.40937	0.10758	21.7	23.5	25.7	28.4	31.9	36.5	43.1
17	:	5	209	-1.29412	28.48021	0.10737	21.8	23.6	25.8	28.5	32.0	36.6	43.2
17	:	6	210	-1.30165	28.54989	0.10716	21.8	23.6	25.8	28.5	32.0	36.7	43.3
17	:	7	211	-1.30865	28.61841	0.10697	21.9	23.7	25.9	28.6	32.1	36.8	43.4
17	:	8	212	-1.31504	28.68577	0.10679	22.0	23.8	26.0	28.7	32.2	36.9	43.5
17	:	9	213	-1.32075	28.75198	0.10661	22.0	23.8	26.0	28.8	32.3	36.9	43.6
17	:	10	214	-1.32576	28.81703	0.10645	22.1	23.9	26.1	28.8	32.3	37.0	43.7
17	:	11	215	-1.33005	28.88093	0.10629	22.1	24.0	26.1	28.9	32.4	37.1	43.7
18	:	0	216	-1.33359	28.94368	0.10614	22.2	24.0	26.2	28.9	32.5	37.1	43.8
18	:	1	217	-1.33637	29.00529	0.10600	22.3	24.1	26.3	29.0	32.5	37.2	43.9
18	:	2	218	-1.33836	29.06574	0.10587	22.3	24.1	26.3	29.1	32.6	37.3	44.0
18	:	3	219	-1.33955	29.12505	0.10575	22.4	24.2	26.4	29.1	32.6	37.3	44.0
18	:	4	220	-1.33992	29.18321	0.10563	22.4	24.2	26.4	29.2	32.7	37.4	44.1
18	:	5	221	-1.33944	29.24023	0.10552	22.5	24.3	26.5	29.2	32.8	37.5	44.1
18	:	6	222	-1.33810	29.29611	0.10542	22.5	24.3	26.5	29.3	32.8	37.5	44.2
18	:	7	223	-1.33587	29.35084	0.10533	22.5	24.4	26.6	29.4	32.9	37.6	44.2
18	:	8	224	-1.33274	29.40443	0.10524	22.6	24.4	26.6	29.4	32.9	37.6	44.3
18	:	9	225	-1.32869	29.45689	0.10516	22.6	24.5	26.7	29.5	33.0	37.7	44.3
18	:	10	226	-1.32369	29.50820	0.10508	22.7	24.5	26.7	29.5	33.0	37.7	44.4
18	:	11	227	-1.31773	29.55838	0.10501	22.7	24.6	26.8	29.6	33.1	37.8	44.4
19	:	0	228	-1.31080	29.60742	0.10495	22.7	24.6	26.8	29.6	33.1	37.8	44.4

Mid-upper arm circumference-for-age GIRLS (5 to 19 years)

5 to 19 years					Z Scores (MUAC in cm)						
Year:Month	Months	L	M	S	-3	-2	-1	0	+1	+2	+3
5 : 0	60	-0.49271	16.81684	0.09338	12.9	14.1	15.3	16.8	18.5	20.5	22.7
5 : 1	61	-0.50464	16.86234	0.09380	13.0	14.1	15.4	16.9	18.6	20.5	22.8
5 : 2	62	-0.51656	16.90778	0.09423	13.0	14.1	15.4	16.9	18.6	20.6	23.0
5 : 3	63	-0.52844	16.95319	0.09467	13.0	14.2	15.5	17.0	18.7	20.7	23.1
5 : 4	64	-0.54030	16.99859	0.09512	13.0	14.2	15.5	17.0	18.7	20.8	23.2
5 : 5	65	-0.55212	17.04400	0.09557	13.1	14.2	15.5	17.0	18.8	20.9	23.3
5 : 6	66	-0.56392	17.08945	0.09603	13.1	14.2	15.6	17.1	18.9	20.9	23.4
5 : 7	67	-0.57568	17.13499	0.09649	13.1	14.3	15.6	17.1	18.9	21.0	23.5
5 : 8	68	-0.58740	17.18065	0.09696	13.1	14.3	15.6	17.2	19.0	21.1	23.6
5 : 9	69	-0.59908	17.22647	0.09744	13.2	14.3	15.7	17.2	19.0	21.2	23.8
5 : 10	70	-0.61073	17.27250	0.09793	13.2	14.4	15.7	17.3	19.1	21.3	23.9
5 : 11	71	-0.62233	17.31876	0.09842	13.2	14.4	15.7	17.3	19.2	21.4	24.0
6 : 0	72	-0.63388	17.36531	0.09892	13.2	14.4	15.8	17.4	19.2	21.5	24.1
6 : 1	73	-0.64539	17.41218	0.09943	13.3	14.4	15.8	17.4	19.3	21.5	24.3
6 : 2	74	-0.65684	17.45942	0.09995	13.3	14.5	15.8	17.5	19.4	21.6	24.4
6 : 3	75	-0.66825	17.50705	0.10047	13.3	14.5	15.9	17.5	19.4	21.7	24.5
6 : 4	76	-0.67959	17.55514	0.10100	13.3	14.5	15.9	17.6	19.5	21.8	24.6
6 : 5	77	-0.69089	17.60370	0.10154	13.4	14.6	16.0	17.6	19.6	21.9	24.8
6 : 6	78	-0.70212	17.65279	0.10208	13.4	14.6	16.0	17.7	19.6	22.0	24.9
6 : 7	79	-0.71329	17.70244	0.10263	13.4	14.6	16.0	17.7	19.7	22.1	25.1
6 : 8	80	-0.72439	17.75270	0.10319	13.4	14.6	16.1	17.8	19.8	22.2	25.2
6 : 9	81	-0.73543	17.80359	0.10375	13.5	14.7	16.1	17.8	19.8	22.3	25.4
6 : 10	82	-0.74641	17.85514	0.10433	13.5	14.7	16.1	17.9	19.9	22.4	25.5
6 : 11	83	-0.75731	17.90734	0.10490	13.5	14.7	16.2	17.9	20.0	22.5	25.7
7 : 0	84	-0.76814	17.96022	0.10549	13.5	14.8	16.2	18.0	20.0	22.6	25.8
7 : 1	85	-0.77891	18.01378	0.10608	13.6	14.8	16.3	18.0	20.1	22.7	26.0
7 : 2	86	-0.78960	18.06804	0.10667	13.6	14.8	16.3	18.1	20.2	22.8	26.1
7 : 3	87	-0.80022	18.12300	0.10727	13.6	14.9	16.4	18.1	20.3	22.9	26.3
7 : 4	88	-0.81076	18.17868	0.10788	13.6	14.9	16.4	18.2	20.4	23.0	26.5
7 : 5	89	-0.82123	18.23510	0.10848	13.7	14.9	16.4	18.2	20.4	23.2	26.6
7 : 6	90	-0.83162	18.29225	0.10909	13.7	15.0	16.5	18.3	20.5	23.3	26.8
7 : 7	91	-0.84193	18.35016	0.10971	13.7	15.0	16.5	18.4	20.6	23.4	27.0
7 : 8	92	-0.85217	18.40883	0.11033	13.8	15.0	16.6	18.4	20.7	23.5	27.2
7 : 9	93	-0.86233	18.46827	0.11094	13.8	15.1	16.6	18.5	20.8	23.6	27.3
7 : 10	94	-0.87240	18.52850	0.11157	13.8	15.1	16.7	18.5	20.8	23.7	27.5
7 : 11	95	-0.88239	18.58953	0.11219	13.8	15.1	16.7	18.6	20.9	23.9	27.7
8 : 0	96	-0.89230	18.65136	0.11281	13.9	15.2	16.8	18.7	21.0	24.0	27.9
8 : 1	97	-0.90213	18.71396	0.11343	13.9	15.2	16.8	18.7	21.1	24.1	28.1
8 : 2	98	-0.91187	18.77734	0.11405	13.9	15.3	16.8	18.8	21.2	24.2	28.3
8 : 3	99	-0.92153	18.84147	0.11467	14.0	15.3	16.9	18.8	21.3	24.4	28.5
8 : 4	100	-0.93111	18.90633	0.11529	14.0	15.3	16.9	18.9	21.4	24.5	28.7
8 : 5	101	-0.94061	18.97192	0.11590	14.0	15.4	17.0	19.0	21.4	24.6	28.9
8 : 6	102	-0.95002	19.03822	0.11651	14.1	15.4	17.0	19.0	21.5	24.8	29.1
8 : 7	103	-0.95935	19.10522	0.11712	14.1	15.5	17.1	19.1	21.6	24.9	29.3
8 : 8	104	-0.96860	19.17289	0.11772	14.2	15.5	17.2	19.2	21.7	25.0	29.5
8 : 9	105	-0.97776	19.24123	0.11831	14.2	15.6	17.2	19.2	21.8	25.2	29.8
8 : 10	106	-0.98685	19.31023	0.11890	14.2	15.6	17.3	19.3	21.9	25.3	30.0
8 : 11	107	-0.99585	19.37986	0.11948	14.3	15.6	17.3	19.4	22.0	25.5	30.2
9 : 0	108	-1.00477	19.45011	0.12006	14.3	15.7	17.4	19.5	22.1	25.6	30.4
9 : 1	109	-1.01360	19.52097	0.12062	14.3	15.7	17.4	19.5	22.2	25.7	30.6
9 : 2	110	-1.02236	19.59243	0.12117	14.4	15.8	17.5	19.6	22.3	25.9	30.9
9 : 3	111	-1.03103	19.66448	0.12171	14.4	15.8	17.5	19.7	22.4	26.0	31.1
9 : 4	112	-1.03963	19.73712	0.12224	14.5	15.9	17.6	19.7	22.5	26.2	31.3
9 : 5	113	-1.04814	19.81035	0.12276	14.5	15.9	17.6	19.8	22.6	26.3	31.6
9 : 6	114	-1.05657	19.88418	0.12327	14.6	16.0	17.7	19.9	22.7	26.5	31.8
9 : 7	115	-1.06493	19.95861	0.12376	14.6	16.0	17.8	20.0	22.8	26.6	32.0
9 : 8	116	-1.07321	20.03364	0.12424	14.6	16.1	17.8	20.0	22.9	26.7	32.2
9 : 9	117	-1.08141	20.10928	0.12471	14.7	16.1	17.9	20.1	23.0	26.9	32.5

5 to 19 years					Z Scores (MUAC in cm)						
Year:Month	Months	L	M	S	-3	-2	-1	0	+1	+2	+3
9 : 10	118	-1.08954	20.18551	0.12515	14.7	16.2	18.0	20.2	23.1	27.0	32.7
9 : 11	119	-1.09759	20.26235	0.12559	14.8	16.2	18.0	20.3	23.2	27.2	32.9
10 : 0	120	-1.10557	20.33979	0.12601	14.8	16.3	18.1	20.3	23.3	27.3	33.2
10 : 1	121	-1.11347	20.41785	0.12641	14.9	16.3	18.1	20.4	23.4	27.5	33.4
10 : 2	122	-1.12131	20.49651	0.12679	14.9	16.4	18.2	20.5	23.5	27.6	33.7
10 : 3	123	-1.12907	20.57578	0.12716	15.0	16.5	18.3	20.6	23.6	27.8	33.9
10 : 4	124	-1.13676	20.65567	0.12751	15.0	16.5	18.3	20.7	23.7	27.9	34.1
10 : 5	125	-1.14439	20.73616	0.12783	15.1	16.6	18.4	20.7	23.8	28.1	34.4
10 : 6	126	-1.15194	20.81724	0.12814	15.1	16.6	18.5	20.8	23.9	28.2	34.6
10 : 7	127	-1.15943	20.89887	0.12843	15.2	16.7	18.5	20.9	24.0	28.4	34.8
10 : 8	128	-1.16685	20.98103	0.12870	15.3	16.8	18.6	21.0	24.1	28.5	35.1
10 : 9	129	-1.17420	21.06370	0.12895	15.3	16.8	18.7	21.1	24.2	28.6	35.3
10 : 10	130	-1.18149	21.14683	0.12918	15.4	16.9	18.8	21.1	24.3	28.8	35.5
10 : 11	131	-1.18871	21.23041	0.12939	15.4	16.9	18.8	21.2	24.4	28.9	35.7
11 : 0	132	-1.19587	21.31441	0.12958	15.5	17.0	18.9	21.3	24.5	29.1	36.0
11 : 1	133	-1.20297	21.39880	0.12976	15.5	17.1	19.0	21.4	24.6	29.2	36.2
11 : 2	134	-1.21000	21.48355	0.12991	15.6	17.1	19.0	21.5	24.7	29.3	36.4
11 : 3	135	-1.21697	21.56863	0.13004	15.7	17.2	19.1	21.6	24.8	29.5	36.6
11 : 4	136	-1.22389	21.65401	0.13016	15.7	17.3	19.2	21.7	25.0	29.6	36.8
11 : 5	137	-1.23074	21.73968	0.13025	15.8	17.3	19.3	21.7	25.1	29.8	37.0
11 : 6	138	-1.23753	21.82559	0.13032	15.9	17.4	19.3	21.8	25.2	29.9	37.2
11 : 7	139	-1.24427	21.91173	0.13038	15.9	17.5	19.4	21.9	25.3	30.0	37.4
11 : 8	140	-1.25095	21.99804	0.13041	16.0	17.6	19.5	22.0	25.4	30.2	37.6
11 : 9	141	-1.25757	22.08447	0.13043	16.1	17.6	19.6	22.1	25.5	30.3	37.8
11 : 10	142	-1.26414	22.17095	0.13043	16.1	17.7	19.6	22.2	25.6	30.4	38.0
11 : 11	143	-1.27064	22.25742	0.13041	16.2	17.8	19.7	22.3	25.7	30.6	38.2
12 : 0	144	-1.27709	22.34380	0.13037	16.3	17.8	19.8	22.3	25.8	30.7	38.4
12 : 1	145	-1.28349	22.43004	0.13032	16.3	17.9	19.9	22.4	25.9	30.8	38.6
12 : 2	146	-1.28983	22.51607	0.13025	16.4	18.0	20.0	22.5	26.0	30.9	38.8
12 : 3	147	-1.29611	22.60182	0.13017	16.5	18.1	20.0	22.6	26.1	31.1	39.0
12 : 4	148	-1.30233	22.68723	0.13008	16.5	18.1	20.1	22.7	26.2	31.2	39.1
12 : 5	149	-1.30850	22.77224	0.12997	16.6	18.2	20.2	22.8	26.3	31.3	39.3
12 : 6	150	-1.31461	22.85678	0.12985	16.7	18.3	20.3	22.9	26.4	31.4	39.5
12 : 7	151	-1.32067	22.94077	0.12971	16.8	18.4	20.4	22.9	26.4	31.5	39.6
12 : 8	152	-1.32667	23.02417	0.12957	16.8	18.4	20.4	23.0	26.5	31.6	39.8
12 : 9	153	-1.33261	23.10690	0.12941	16.9	18.5	20.5	23.1	26.6	31.7	39.9
12 : 10	154	-1.33850	23.18890	0.12925	17.0	18.6	20.6	23.2	26.7	31.8	40.1
12 : 11	155	-1.34434	23.27013	0.12907	17.0	18.6	20.7	23.3	26.8	32.0	40.2
13 : 0	156	-1.35012	23.35053	0.12889	17.1	18.7	20.7	23.4	26.9	32.1	40.3
13 : 1	157	-1.35584	23.43008	0.12870	17.2	18.8	20.8	23.4	27.0	32.2	40.5
13 : 2	158	-1.36150	23.50874	0.12850	17.2	18.9	20.9	23.5	27.1	32.3	40.6
13 : 3	159	-1.36711	23.58647	0.12830	17.3	18.9	21.0	23.6	27.2	32.4	40.7
13 : 4	160	-1.37265	23.66323	0.12809	17.4	19.0	21.0	23.7	27.2	32.4	40.9
13 : 5	161	-1.37814	23.73898	0.12787	17.4	19.1	21.1	23.7	27.3	32.5	41.0
13 : 6	162	-1.38357	23.81369	0.12766	17.5	19.1	21.2	23.8	27.4	32.6	41.1
13 : 7	163	-1.38894	23.88732	0.12744	17.6	19.2	21.2	23.9	27.5	32.7	41.2
13 : 8	164	-1.39425	23.95982	0.12721	17.6	19.3	21.3	24.0	27.6	32.8	41.3
13 : 9	165	-1.39950	24.03118	0.12699	17.7	19.3	21.4	24.0	27.6	32.9	41.4
13 : 10	166	-1.40469	24.10133	0.12676	17.8	19.4	21.4	24.1	27.7	33.0	41.5
13 : 11	167	-1.40982	24.17025	0.12653	17.8	19.5	21.5	24.2	27.8	33.1	41.6
14 : 0	168	-1.41489	24.23790	0.12630	17.9	19.5	21.6	24.2	27.9	33.1	41.7
14 : 1	169	-1.41989	24.30425	0.12608	18.0	19.6	21.6	24.3	27.9	33.2	41.8
14 : 2	170	-1.42484	24.36928	0.12585	18.0	19.7	21.7	24.4	28.0	33.3	41.9
14 : 3	171	-1.42971	24.43300	0.12563	18.1	19.7	21.8	24.4	28.1	33.4	42.0
14 : 4	172	-1.43453	24.49541	0.12541	18.1	19.8	21.8	24.5	28.1	33.4	42.1
14 : 5	173	-1.43928	24.55649	0.12519	18.2	19.8	21.9	24.6	28.2	33.5	42.2
14 : 6	174	-1.44397	24.61626	0.12498	18.2	19.9	21.9	24.6	28.3	33.6	42.2
14 : 7	175	-1.44859	24.67470	0.12476	18.3	19.9	22.0	24.7	28.3	33.6	42.3
14 : 8	176	-1.45315	24.73182	0.12456	18.3	20.0	22.1	24.7	28.4	33.7	42.4

5 to 19 years								Z Scores (MUAC in cm)			
Year:Month	Months	L	M	S	-3	-2	-1	0	+1	+2	+3
14 : 9	177	-1.45764	24.78761	0.12436	18.4	20.0	22.1	24.8	28.4	33.8	42.5
14 : 10	178	-1.46207	24.84208	0.12416	18.5	20.1	22.2	24.8	28.5	33.8	42.5
14 : 11	179	-1.46642	24.89521	0.12397	18.5	20.1	22.2	24.9	28.5	33.9	42.6
15 : 0	180	-1.47072	24.94702	0.12378	18.5	20.2	22.3	24.9	28.6	33.9	42.7
15 : 1	181	-1.47494	24.99749	0.12360	18.6	20.2	22.3	25.0	28.7	34.0	42.8
15 : 2	182	-1.47910	25.04663	0.12343	18.6	20.3	22.4	25.0	28.7	34.1	42.8
15 : 3	183	-1.48319	25.09442	0.12326	18.7	20.3	22.4	25.1	28.8	34.1	42.9
15 : 4	184	-1.48721	25.14089	0.12311	18.7	20.4	22.5	25.1	28.8	34.2	43.0
15 : 5	185	-1.49116	25.18605	0.12296	18.8	20.4	22.5	25.2	28.9	34.2	43.0
15 : 6	186	-1.49504	25.22993	0.12281	18.8	20.5	22.5	25.2	28.9	34.3	43.1
15 : 7	187	-1.49886	25.27255	0.12268	18.9	20.5	22.6	25.3	28.9	34.3	43.2
15 : 8	188	-1.50260	25.31393	0.12255	18.9	20.5	22.6	25.3	29.0	34.4	43.2
15 : 9	189	-1.50628	25.35410	0.12243	18.9	20.6	22.7	25.4	29.0	34.4	43.3
15 : 10	190	-1.50989	25.39309	0.12232	19.0	20.6	22.7	25.4	29.1	34.5	43.4
15 : 11	191	-1.51343	25.43091	0.12222	19.0	20.7	22.7	25.4	29.1	34.5	43.4
16 : 0	192	-1.51691	25.46760	0.12213	19.0	20.7	22.8	25.5	29.2	34.6	43.5
16 : 1	193	-1.52031	25.50317	0.12204	19.1	20.7	22.8	25.5	29.2	34.6	43.5
16 : 2	194	-1.52364	25.53766	0.12196	19.1	20.8	22.8	25.5	29.2	34.6	43.6
16 : 3	195	-1.52691	25.57108	0.12189	19.1	20.8	22.9	25.6	29.3	34.7	43.7
16 : 4	196	-1.53011	25.60346	0.12183	19.2	20.8	22.9	25.6	29.3	34.7	43.7
16 : 5	197	-1.53324	25.63483	0.12177	19.2	20.8	22.9	25.6	29.3	34.8	43.8
16 : 6	198	-1.53630	25.66520	0.12173	19.2	20.9	23.0	25.7	29.4	34.8	43.9
16 : 7	199	-1.53930	25.69462	0.12169	19.2	20.9	23.0	25.7	29.4	34.9	43.9
16 : 8	200	-1.54222	25.72310	0.12166	19.3	20.9	23.0	25.7	29.4	34.9	44.0
16 : 9	201	-1.54508	25.75069	0.12164	19.3	20.9	23.0	25.8	29.5	34.9	44.1
16 : 10	202	-1.54788	25.77741	0.12163	19.3	21.0	23.1	25.8	29.5	35.0	44.1
16 : 11	203	-1.55061	25.80329	0.12162	19.3	21.0	23.1	25.8	29.5	35.0	44.2
17 : 0	204	-1.55328	25.82837	0.12162	19.3	21.0	23.1	25.8	29.6	35.1	44.3
17 : 1	205	-1.55588	25.85269	0.12163	19.4	21.0	23.1	25.9	29.6	35.1	44.3
17 : 2	206	-1.55843	25.87626	0.12165	19.4	21.1	23.1	25.9	29.6	35.1	44.4
17 : 3	207	-1.56091	25.89913	0.12167	19.4	21.1	23.2	25.9	29.6	35.2	44.5
17 : 4	208	-1.56333	25.92133	0.12170	19.4	21.1	23.2	25.9	29.7	35.2	44.5
17 : 5	209	-1.56569	25.94288	0.12174	19.4	21.1	23.2	25.9	29.7	35.3	44.6
17 : 6	210	-1.56799	25.96382	0.12178	19.5	21.1	23.2	26.0	29.7	35.3	44.7
17 : 7	211	-1.57024	25.98419	0.12183	19.5	21.1	23.2	26.0	29.7	35.3	44.7
17 : 8	212	-1.57243	26.00401	0.12189	19.5	21.2	23.3	26.0	29.8	35.4	44.8
17 : 9	213	-1.57456	26.02332	0.12195	19.5	21.2	23.3	26.0	29.8	35.4	44.9
17 : 10	214	-1.57664	26.04213	0.12202	19.5	21.2	23.3	26.0	29.8	35.4	45.0
17 : 11	215	-1.57867	26.06047	0.12210	19.5	21.2	23.3	26.1	29.8	35.5	45.0
18 : 0	216	-1.58064	26.07835	0.12218	19.5	21.2	23.3	26.1	29.9	35.5	45.1
18 : 1	217	-1.58256	26.09580	0.12227	19.5	21.2	23.3	26.1	29.9	35.6	45.2
18 : 2	218	-1.58443	26.11283	0.12236	19.6	21.2	23.3	26.1	29.9	35.6	45.3
18 : 3	219	-1.58625	26.12947	0.12246	19.6	21.2	23.4	26.1	29.9	35.6	45.3
18 : 4	220	-1.58803	26.14572	0.12256	19.6	21.3	23.4	26.1	30.0	35.7	45.4
18 : 5	221	-1.58975	26.16161	0.12267	19.6	21.3	23.4	26.2	30.0	35.7	45.5
18 : 6	222	-1.59143	26.17717	0.12278	19.6	21.3	23.4	26.2	30.0	35.7	45.6
18 : 7	223	-1.59307	26.19240	0.12290	19.6	21.3	23.4	26.2	30.0	35.8	45.7
18 : 8	224	-1.59466	26.20732	0.12302	19.6	21.3	23.4	26.2	30.1	35.8	45.7
18 : 9	225	-1.59621	26.22197	0.12315	19.6	21.3	23.4	26.2	30.1	35.9	45.8
18 : 10	226	-1.59771	26.23634	0.12328	19.6	21.3	23.4	26.2	30.1	35.9	45.9
18 : 11	227	-1.59918	26.25047	0.12342	19.6	21.3	23.5	26.3	30.1	35.9	46.0
19 : 0	228	-1.60061	26.26437	0.12356	19.6	21.3	23.5	26.3	30.1	36.0	46.1

Mid-upper arm circumference-for-age BOYS. (5 to 19 years)

5 to 19 years					Percentiles (MUAC in cm)										
Year : Month	Months	L	M	S	1st	3rd	5th	15th	25th	50th	75th	85th	95th	97th	99th
5 : 0	60	-0.72895	16.5189	0.08670	13.7	14.2	14.4	15.1	15.6	16.5	17.5	18.1	19.2	19.7	20.5
5 : 1	61	-0.75845	16.5516	0.08699	13.7	14.2	14.4	15.2	15.6	16.6	17.6	18.2	19.3	19.7	20.6
5 : 2	62	-0.78927	16.5856	0.08728	13.7	14.2	14.5	15.2	15.7	16.6	17.6	18.2	19.3	19.8	20.7
5 : 3	63	-0.82138	16.6212	0.08758	13.8	14.2	14.5	15.2	15.7	16.6	17.7	18.3	19.4	19.8	20.8
5 : 4	64	-0.85473	16.6585	0.08789	13.8	14.3	14.5	15.3	15.7	16.7	17.7	18.3	19.4	19.9	20.9
5 : 5	65	-0.88928	16.6978	0.08819	13.8	14.3	14.6	15.3	15.8	16.7	17.7	18.4	19.5	20.0	20.9
5 : 6	66	-0.92496	16.7391	0.08851	13.9	14.3	14.6	15.3	15.8	16.7	17.8	18.4	19.6	20.1	21.0
5 : 7	67	-0.96164	16.7825	0.08882	13.9	14.4	14.6	15.4	15.8	16.8	17.8	18.5	19.6	20.1	21.1
5 : 8	68	-0.99919	16.8277	0.08915	13.9	14.4	14.7	15.4	15.9	16.8	17.9	18.5	19.7	20.2	21.2
5 : 9	69	-1.03745	16.8746	0.08948	14.0	14.4	14.7	15.4	15.9	16.9	18.0	18.6	19.8	20.3	21.3
5 : 10	70	-1.07629	16.9232	0.08981	14.0	14.5	14.8	15.5	16.0	16.9	18.0	18.7	19.9	20.4	21.4
5 : 11	71	-1.11556	16.9732	0.09015	14.1	14.5	14.8	15.5	16.0	17.0	18.1	18.7	20.0	20.5	21.6
6 : 0	72	-1.15511	17.0247	0.09049	14.1	14.6	14.8	15.6	16.1	17.0	18.1	18.8	20.0	20.6	21.7
6 : 1	73	-1.19480	17.0773	0.09084	14.1	14.6	14.9	15.6	16.1	17.1	18.2	18.9	20.1	20.7	21.8
6 : 2	74	-1.23449	17.1311	0.09120	14.2	14.7	14.9	15.7	16.1	17.1	18.3	18.9	20.2	20.8	21.9
6 : 3	75	-1.27403	17.1858	0.09156	14.2	14.7	15.0	15.7	16.2	17.2	18.3	19.0	20.3	20.9	22.0
6 : 4	76	-1.31328	17.2414	0.09193	14.3	14.8	15.0	15.8	16.2	17.2	18.4	19.1	20.4	21.0	22.2
6 : 5	77	-1.35210	17.2977	0.09231	14.3	14.8	15.1	15.8	16.3	17.3	18.5	19.2	20.5	21.1	22.3
6 : 6	78	-1.39034	17.3547	0.09269	14.4	14.8	15.1	15.9	16.3	17.4	18.5	19.2	20.6	21.2	22.4
6 : 7	79	-1.42785	17.4121	0.09308	14.4	14.9	15.2	15.9	16.4	17.4	18.6	19.3	20.7	21.3	22.6
6 : 8	80	-1.46450	17.4698	0.09347	14.5	14.9	15.2	16.0	16.4	17.5	18.7	19.4	20.8	21.4	22.7
6 : 9	81	-1.50018	17.5278	0.09388	14.5	15.0	15.3	16.0	16.5	17.5	18.7	19.5	20.9	21.5	22.8
6 : 10	82	-1.53485	17.5861	0.09428	14.6	15.0	15.3	16.1	16.6	17.6	18.8	19.5	21.0	21.6	23.0
6 : 11	83	-1.56850	17.6445	0.09470	14.6	15.1	15.3	16.1	16.6	17.6	18.9	19.6	21.1	21.7	23.1
7 : 0	84	-1.60108	17.7031	0.09512	14.6	15.1	15.4	16.2	16.7	17.7	18.9	19.7	21.2	21.9	23.3
7 : 1	85	-1.63258	17.7618	0.09555	14.7	15.2	15.4	16.2	16.7	17.8	19.0	19.8	21.3	22.0	23.4
7 : 2	86	-1.66297	17.8206	0.09598	14.7	15.2	15.5	16.3	16.8	17.8	19.1	19.9	21.4	22.1	23.6
7 : 3	87	-1.69222	17.8795	0.09642	14.8	15.3	15.5	16.3	16.8	17.9	19.2	19.9	21.5	22.2	23.7
7 : 4	88	-1.72030	17.9384	0.09687	14.8	15.3	15.6	16.4	16.9	17.9	19.2	20.0	21.6	22.3	23.9
7 : 5	89	-1.74719	17.9973	0.09732	14.9	15.4	15.6	16.4	16.9	18.0	19.3	20.1	21.7	22.4	24.0
7 : 6	90	-1.77285	18.0562	0.09778	14.9	15.4	15.7	16.4	17.0	18.1	19.4	20.2	21.8	22.6	24.2
7 : 7	91	-1.79727	18.1150	0.09824	15.0	15.4	15.7	16.5	17.0	18.1	19.4	20.3	21.9	22.7	24.3
7 : 8	92	-1.82040	18.1737	0.09871	15.0	15.5	15.8	16.5	17.1	18.2	19.5	20.4	22.0	22.8	24.5
7 : 9	93	-1.84224	18.2323	0.09918	15.0	15.5	15.8	16.6	17.1	18.2	19.6	20.4	22.1	22.9	24.6
7 : 10	94	-1.86274	18.2908	0.09966	15.1	15.6	15.9	16.6	17.2	18.3	19.7	20.5	22.2	23.0	24.8
7 : 11	95	-1.88189	18.3491	0.10014	15.1	15.6	15.9	16.7	17.2	18.3	19.7	20.6	22.3	23.2	24.9
8 : 0	96	-1.89968	18.4072	0.10063	15.2	15.7	15.9	16.7	17.3	18.4	19.8	20.7	22.5	23.3	25.1
8 : 1	97	-1.91614	18.4652	0.10112	15.2	15.7	16.0	16.8	17.3	18.5	19.9	20.8	22.6	23.4	25.2
8 : 2	98	-1.93128	18.5232	0.10162	15.2	15.7	16.0	16.8	17.4	18.5	19.9	20.8	22.7	23.5	25.4
8 : 3	99	-1.94514	18.5813	0.10211	15.3	15.8	16.1	16.9	17.4	18.6	20.0	20.9	22.8	23.6	25.6
8 : 4	100	-1.95773	18.6394	0.10262	15.3	15.8	16.1	16.9	17.5	18.6	20.1	21.0	22.9	23.8	25.7
8 : 5	101	-1.96908	18.6977	0.10312	15.4	15.9	16.2	17.0	17.5	18.7	20.1	21.1	23.0	23.9	25.9
8 : 6	102	-1.97920	18.7561	0.10363	15.4	15.9	16.2	17.0	17.6	18.8	20.2	21.2	23.1	24.0	26.0
8 : 7	103	-1.98813	18.8149	0.10414	15.4	15.9	16.2	17.1	17.6	18.8	20.3	21.2	23.2	24.1	26.2
8 : 8	104	-1.99588	18.8740	0.10465	15.5	16.0	16.3	17.1	17.7	18.9	20.4	21.3	23.3	24.2	26.3
8 : 9	105	-2.00248	18.9335	0.10516	15.5	16.0	16.3	17.2	17.7	18.9	20.4	21.4	23.4	24.4	26.5
8 : 10	106	-2.00795	18.9934	0.10568	15.6	16.1	16.4	17.2	17.8	19.0	20.5	21.5	23.5	24.5	26.7
8 : 11	107	-2.01231	19.0539	0.10619	15.6	16.1	16.4	17.3	17.8	19.1	20.6	21.6	23.6	24.6	26.8
9 : 0	108	-2.01558	19.1149	0.10671	15.6	16.2	16.4	17.3	17.9	19.1	20.7	21.7	23.7	24.7	27.0
9 : 1	109	-2.01780	19.1766	0.10722	15.7	16.2	16.5	17.3	17.9	19.2	20.7	21.7	23.8	24.8	27.1
9 : 2	110	-2.01897	19.2391	0.10774	15.7	16.2	16.5	17.4	18.0	19.2	20.8	21.8	24.0	25.0	27.3
9 : 3	111	-2.01912	19.3022	0.10825	15.7	16.3	16.6	17.4	18.0	19.3	20.9	21.9	24.1	25.1	27.4
9 : 4	112	-2.01828	19.3661	0.10877	15.8	16.3	16.6	17.5	18.1	19.4	21.0	22.0	24.2	25.2	27.6
9 : 5	113	-2.01647	19.4309	0.10928	15.8	16.4	16.7	17.5	18.1	19.4	21.0	22.1	24.3	25.3	27.7
9 : 6	114	-2.01371	19.4965	0.10979	15.9	16.4	16.7	17.6	18.2	19.5	21.1	22.2	24.4	25.5	27.9
9 : 7	115	-2.01004	19.5629	0.11029	15.9	16.4	16.8	17.7	18.3	19.6	21.2	22.3	24.5	25.6	28.1
9 : 8	116	-2.00546	19.6303	0.11080	15.9	16.5	16.8	17.7	18.3	19.6	21.3	22.4	24.6	25.7	28.2
9 : 9	117	-2.00000	19.6986	0.11130	16.0	16.5	16.9	17.8	18.4	19.7	21.4	22.5	24.7	25.8	28.4

5 to 19 years					Percentiles (MUAC in cm)										
Year : Month	Months	L	M	S	1st	3rd	5th	15th	25th	50th	75th	85th	95th	97th	99th
9 : 10	118	-1.99370	19.7679	0.11179	16.0	16.6	16.9	17.8	18.4	19.8	21.4	22.6	24.9	26.0	28.5
9 : 11	119	-1.98657	19.8383	0.11228	16.1	16.6	17.0	17.9	18.5	19.8	21.5	22.6	25.0	26.1	28.7
10 : 0	120	-1.97863	19.9096	0.11277	16.1	16.7	17.0	17.9	18.5	19.9	21.6	22.7	25.1	26.2	28.8
10 : 1	121	-1.96991	19.9821	0.11325	16.2	16.7	17.0	18.0	18.6	20.0	21.7	22.8	25.2	26.3	29.0
10 : 2	122	-1.96043	20.0556	0.11372	16.2	16.8	17.1	18.0	18.7	20.1	21.8	22.9	25.3	26.5	29.1
10 : 3	123	-1.95022	20.1303	0.11419	16.3	16.8	17.2	18.1	18.7	20.1	21.9	23.0	25.4	26.6	29.3
10 : 4	124	-1.93930	20.2062	0.11465	16.3	16.9	17.2	18.2	18.8	20.2	22.0	23.1	25.6	26.7	29.4
10 : 5	125	-1.92770	20.2833	0.11510	16.3	16.9	17.3	18.2	18.9	20.3	22.1	23.2	25.7	26.8	29.6
10 : 6	126	-1.91544	20.3617	0.11554	16.4	17.0	17.3	18.3	18.9	20.4	22.2	23.3	25.8	27.0	29.7
10 : 7	127	-1.90255	20.4412	0.11597	16.4	17.0	17.4	18.3	19.0	20.4	22.2	23.4	25.9	27.1	29.8
10 : 8	128	-1.88906	20.5220	0.11640	16.5	17.1	17.4	18.4	19.1	20.5	22.3	23.5	26.0	27.2	30.0
10 : 9	129	-1.87501	20.6041	0.11681	16.5	17.1	17.5	18.5	19.1	20.6	22.4	23.6	26.1	27.3	30.1
10 : 10	130	-1.86041	20.6874	0.11721	16.6	17.2	17.5	18.5	19.2	20.7	22.5	23.7	26.3	27.5	30.3
10 : 11	131	-1.84530	20.7720	0.11761	16.6	17.3	17.6	18.6	19.3	20.8	22.6	23.8	26.4	27.6	30.4
11 : 0	132	-1.82971	20.8578	0.11798	16.7	17.3	17.7	18.7	19.4	20.9	22.7	24.0	26.5	27.7	30.5
11 : 1	133	-1.81367	20.9450	0.11835	16.8	17.4	17.7	18.7	19.4	20.9	22.8	24.1	26.6	27.9	30.7
11 : 2	134	-1.79720	21.0334	0.11870	16.8	17.4	17.8	18.8	19.5	21.0	22.9	24.2	26.8	28.0	30.8
11 : 3	135	-1.78034	21.1232	0.11904	16.9	17.5	17.9	18.9	19.6	21.1	23.0	24.3	26.9	28.1	30.9
11 : 4	136	-1.76312	21.2143	0.11937	16.9	17.6	17.9	19.0	19.7	21.2	23.1	24.4	27.0	28.2	31.1
11 : 5	137	-1.74555	21.3067	0.11967	17.0	17.6	18.0	19.0	19.8	21.3	23.2	24.5	27.1	28.4	31.2
11 : 6	138	-1.72768	21.4005	0.11997	17.0	17.7	18.1	19.1	19.8	21.4	23.3	24.6	27.2	28.5	31.3
11 : 7	139	-1.70954	21.4956	0.12024	17.1	17.8	18.1	19.2	19.9	21.5	23.5	24.7	27.4	28.6	31.4
11 : 8	140	-1.69114	21.5921	0.12050	17.2	17.8	18.2	19.3	20.0	21.6	23.6	24.8	27.5	28.7	31.6
11 : 9	141	-1.67254	21.6898	0.12075	17.2	17.9	18.3	19.4	20.1	21.7	23.7	25.0	27.6	28.9	31.7
11 : 10	142	-1.65377	21.7888	0.12097	17.3	18.0	18.3	19.4	20.2	21.8	23.8	25.1	27.7	29.0	31.8
11 : 11	143	-1.63486	21.8890	0.12118	17.4	18.0	18.4	19.5	20.3	21.9	23.9	25.2	27.9	29.1	31.9
12 : 0	144	-1.61586	21.9904	0.12137	17.4	18.1	18.5	19.6	20.4	22.0	24.0	25.3	28.0	29.2	32.1
12 : 1	145	-1.59680	22.0929	0.12154	17.5	18.2	18.6	19.7	20.5	22.1	24.1	25.4	28.1	29.4	32.2
12 : 2	146	-1.57771	22.1965	0.12169	17.6	18.3	18.7	19.8	20.5	22.2	24.2	25.5	28.2	29.5	32.3
12 : 3	147	-1.55864	22.3012	0.12183	17.6	18.3	18.7	19.9	20.6	22.3	24.3	25.7	28.4	29.6	32.4
12 : 4	148	-1.53962	22.4068	0.12194	17.7	18.4	18.8	20.0	20.7	22.4	24.5	25.8	28.5	29.7	32.5
12 : 5	149	-1.52070	22.5134	0.12204	17.8	18.5	18.9	20.1	20.8	22.5	24.6	25.9	28.6	29.9	32.6
12 : 6	150	-1.50190	22.6209	0.12212	17.9	18.6	19.0	20.1	20.9	22.6	24.7	26.0	28.7	30.0	32.8
12 : 7	151	-1.48326	22.7293	0.12218	17.9	18.7	19.1	20.2	21.0	22.7	24.8	26.1	28.9	30.1	32.9
12 : 8	152	-1.46482	22.8385	0.12221	18.0	18.7	19.1	20.3	21.1	22.8	24.9	26.3	29.0	30.2	33.0
12 : 9	153	-1.44663	22.9485	0.12223	18.1	18.8	19.2	20.4	21.2	22.9	25.1	26.4	29.1	30.3	33.1
12 : 10	154	-1.42870	23.0593	0.12223	18.2	18.9	19.3	20.5	21.3	23.1	25.2	26.5	29.2	30.5	33.2
12 : 11	155	-1.41108	23.1707	0.12221	18.2	19.0	19.4	20.6	21.4	23.2	25.3	26.6	29.4	30.6	33.3
13 : 0	156	-1.39378	23.2827	0.12217	18.3	19.1	19.5	20.7	21.5	23.3	25.4	26.8	29.5	30.7	33.4
13 : 1	157	-1.37683	23.3952	0.12211	18.4	19.2	19.6	20.8	21.6	23.4	25.5	26.9	29.6	30.8	33.5
13 : 2	158	-1.36024	23.5082	0.12204	18.5	19.3	19.7	20.9	21.7	23.5	25.7	27.0	29.7	31.0	33.7
13 : 3	159	-1.34404	23.6215	0.12194	18.6	19.3	19.8	21.0	21.8	23.6	25.8	27.1	29.8	31.1	33.8
13 : 4	160	-1.32825	23.7350	0.12183	18.7	19.4	19.9	21.1	22.0	23.7	25.9	27.3	30.0	31.2	33.9
13 : 5	161	-1.31289	23.8488	0.12170	18.7	19.5	20.0	21.2	22.1	23.8	26.0	27.4	30.1	31.3	34.0
13 : 6	162	-1.29798	23.9626	0.12155	18.8	19.6	20.1	21.3	22.2	24.0	26.1	27.5	30.2	31.4	34.1
13 : 7	163	-1.28355	24.0765	0.12138	18.9	19.7	20.2	21.4	22.3	24.1	26.3	27.6	30.3	31.5	34.2
13 : 8	164	-1.26961	24.1903	0.12120	19.0	19.8	20.3	21.5	22.4	24.2	26.4	27.7	30.4	31.7	34.3
13 : 9	165	-1.25619	24.3039	0.12101	19.1	19.9	20.3	21.6	22.5	24.3	26.5	27.9	30.6	31.8	34.4
13 : 10	166	-1.24331	24.4174	0.12079	19.2	20.0	20.4	21.7	22.6	24.4	26.6	28.0	30.7	31.9	34.5
13 : 11	167	-1.23099	24.5305	0.12057	19.3	20.1	20.5	21.8	22.7	24.5	26.7	28.1	30.8	32.0	34.6
14 : 0	168	-1.21926	24.6432	0.12032	19.4	20.2	20.6	21.9	22.8	24.6	26.8	28.2	30.9	32.1	34.7
14 : 1	169	-1.20812	24.7555	0.12007	19.5	20.3	20.7	22.0	22.9	24.8	27.0	28.3	31.0	32.2	34.8
14 : 2	170	-1.19761	24.8672	0.11980	19.6	20.4	20.8	22.2	23.0	24.9	27.1	28.4	31.1	32.3	34.9
14 : 3	171	-1.18774	24.9784	0.11952	19.6	20.5	20.9	22.3	23.1	25.0	27.2	28.6	31.2	32.4	35.0
14 : 4	172	-1.17852	25.0890	0.11922	19.7	20.6	21.0	22.4	23.2	25.1	27.3	28.7	31.4	32.6	35.1
14 : 5	173	-1.16998	25.1989	0.11892	19.8	20.7	21.1	22.5	23.3	25.2	27.4	28.8	31.5	32.7	35.2
14 : 6	174	-1.16213	25.3082	0.11860	19.9	20.8	21.2	22.6	23.4	25.3	27.5	28.9	31.6	32.8	35.3
14 : 7	175	-1.15499	25.4167	0.11828	20.0	20.9	21.3	22.7	23.6	25.4	27.6	29.0	31.7	32.9	35.4
14 : 8	176	-1.14858	25.5245	0.11795	20.1	20.9	21.4	22.8	23.7	25.5	27.7	29.1	31.8	33.0	35.5

5 to 19 years					Percentiles (MUAC in cm)										
Year : Month	Months	L	M	S	1st	3rd	5th	15th	25th	50th	75th	85th	95th	97th	99th
14 : 9	177	-1.14291	25.6314	0.11761	20.2	21.0	21.5	22.9	23.8	25.6	27.9	29.2	31.9	33.1	35.6
14 : 10	178	-1.13800	25.7375	0.11726	20.3	21.1	21.6	23.0	23.9	25.7	28.0	29.3	32.0	33.2	35.7
14 : 11	179	-1.13388	25.8427	0.11691	20.4	21.2	21.7	23.1	24.0	25.8	28.1	29.4	32.1	33.3	35.8
15 : 0	180	-1.13055	25.9470	0.11655	20.5	21.3	21.8	23.2	24.1	25.9	28.2	29.5	32.2	33.4	35.9
15 : 1	181	-1.12804	26.0503	0.11619	20.6	21.4	21.9	23.3	24.2	26.1	28.3	29.6	32.3	33.5	36.0
15 : 2	182	-1.12636	26.1526	0.11583	20.7	21.5	22.0	23.4	24.3	26.2	28.4	29.8	32.4	33.6	36.1
15 : 3	183	-1.12553	26.2538	0.11546	20.8	21.6	22.1	23.5	24.4	26.3	28.5	29.9	32.5	33.7	36.1
15 : 4	184	-1.12555	26.3539	0.11510	20.9	21.7	22.2	23.6	24.5	26.4	28.6	30.0	32.6	33.8	36.2
15 : 5	185	-1.12638	26.4529	0.11473	20.9	21.8	22.3	23.7	24.6	26.5	28.7	30.1	32.7	33.9	36.3
15 : 6	186	-1.12800	26.5508	0.11436	21.0	21.9	22.4	23.8	24.7	26.6	28.8	30.2	32.8	34.0	36.4
15 : 7	187	-1.13037	26.6476	0.11399	21.1	22.0	22.5	23.9	24.8	26.6	28.9	30.2	32.9	34.1	36.5
15 : 8	188	-1.13345	26.7432	0.11362	21.2	22.1	22.6	23.9	24.8	26.7	29.0	30.3	33.0	34.2	36.6
15 : 9	189	-1.13722	26.8377	0.11326	21.3	22.2	22.7	24.0	24.9	26.8	29.1	30.4	33.1	34.3	36.7
15 : 10	190	-1.14163	26.9311	0.11290	21.4	22.3	22.8	24.1	25.0	26.9	29.2	30.5	33.2	34.3	36.8
15 : 11	191	-1.14666	27.0233	0.11254	21.5	22.4	22.8	24.2	25.1	27.0	29.3	30.6	33.3	34.4	36.9
16 : 0	192	-1.15227	27.1144	0.11219	21.6	22.4	22.9	24.3	25.2	27.1	29.3	30.7	33.4	34.5	37.0
16 : 1	193	-1.15843	27.2043	0.11184	21.7	22.5	23.0	24.4	25.3	27.2	29.4	30.8	33.5	34.6	37.1
16 : 2	194	-1.16511	27.2930	0.11150	21.8	22.6	23.1	24.5	25.4	27.3	29.5	30.9	33.5	34.7	37.2
16 : 3	195	-1.17226	27.3805	0.11116	21.8	22.7	23.2	24.6	25.5	27.4	29.6	31.0	33.6	34.8	37.3
16 : 4	196	-1.17986	27.4669	0.11083	21.9	22.8	23.3	24.7	25.6	27.5	29.7	31.1	33.7	34.9	37.4
16 : 5	197	-1.18788	27.5520	0.11051	22.0	22.9	23.4	24.7	25.7	27.6	29.8	31.2	33.8	35.0	37.4
16 : 6	198	-1.19627	27.6359	0.11020	22.1	23.0	23.5	24.8	25.7	27.6	29.9	31.2	33.9	35.1	37.5
16 : 7	199	-1.20499	27.7186	0.10990	22.2	23.0	23.5	24.9	25.8	27.7	30.0	31.3	34.0	35.2	37.6
16 : 8	200	-1.21395	27.8002	0.10960	22.3	23.1	23.6	25.0	25.9	27.8	30.0	31.4	34.1	35.2	37.7
16 : 9	201	-1.22310	27.8805	0.10932	22.3	23.2	23.7	25.1	26.0	27.9	30.1	31.5	34.2	35.3	37.8
16 : 10	202	-1.23235	27.9596	0.10904	22.4	23.3	23.8	25.2	26.1	28.0	30.2	31.6	34.2	35.4	37.9
16 : 11	203	-1.24164	28.0375	0.10878	22.5	23.4	23.9	25.2	26.1	28.0	30.3	31.7	34.3	35.5	38.0
17 : 0	204	-1.25090	28.1142	0.10852	22.6	23.4	23.9	25.3	26.2	28.1	30.4	31.7	34.4	35.6	38.1
17 : 1	205	-1.26004	28.1898	0.10827	22.7	23.5	24.0	25.4	26.3	28.2	30.4	31.8	34.5	35.7	38.2
17 : 2	206	-1.26901	28.2642	0.10803	22.7	23.6	24.1	25.5	26.4	28.3	30.5	31.9	34.6	35.8	38.3
17 : 3	207	-1.27772	28.3374	0.10780	22.8	23.7	24.2	25.5	26.4	28.3	30.6	32.0	34.6	35.8	38.3
17 : 4	208	-1.28612	28.4094	0.10758	22.9	23.7	24.2	25.6	26.5	28.4	30.7	32.0	34.7	35.9	38.4
17 : 5	209	-1.29412	28.4802	0.10737	22.9	23.8	24.3	25.7	26.6	28.5	30.7	32.1	34.8	36.0	38.5
17 : 6	210	-1.30165	28.5499	0.10716	23.0	23.9	24.4	25.7	26.6	28.5	30.8	32.2	34.9	36.1	38.6
17 : 7	211	-1.30865	28.6184	0.10697	23.1	23.9	24.4	25.8	26.7	28.6	30.9	32.3	35.0	36.1	38.7
17 : 8	212	-1.31504	28.6858	0.10679	23.1	24.0	24.5	25.9	26.8	28.7	30.9	32.3	35.0	36.2	38.7
17 : 9	213	-1.32075	28.7520	0.10661	23.2	24.1	24.6	25.9	26.8	28.8	31.0	32.4	35.1	36.3	38.8
17 : 10	214	-1.32576	28.8170	0.10645	23.3	24.1	24.6	26.0	26.9	28.8	31.1	32.5	35.2	36.4	38.9
17 : 11	215	-1.33005	28.8809	0.10629	23.3	24.2	24.7	26.1	27.0	28.9	31.1	32.5	35.2	36.4	39.0
18 : 0	216	-1.33359	28.9437	0.10614	23.4	24.2	24.7	26.1	27.0	28.9	31.2	32.6	35.3	36.5	39.0
18 : 1	217	-1.33637	29.0053	0.10600	23.4	24.3	24.8	26.2	27.1	29.0	31.3	32.7	35.4	36.6	39.1
18 : 2	218	-1.33836	29.0657	0.10587	23.5	24.4	24.9	26.2	27.2	29.1	31.3	32.7	35.4	36.6	39.2
18 : 3	219	-1.33955	29.1250	0.10575	23.5	24.4	24.9	26.3	27.2	29.1	31.4	32.8	35.5	36.7	39.3
18 : 4	220	-1.33992	29.1832	0.10563	23.6	24.5	25.0	26.4	27.3	29.2	31.5	32.8	35.6	36.8	39.3
18 : 5	221	-1.33944	29.2402	0.10552	23.6	24.5	25.0	26.4	27.3	29.2	31.5	32.9	35.6	36.8	39.4
18 : 6	222	-1.33810	29.2961	0.10542	23.7	24.6	25.1	26.5	27.4	29.3	31.6	33.0	35.7	36.9	39.4
18 : 7	223	-1.33587	29.3508	0.10533	23.7	24.6	25.1	26.5	27.4	29.4	31.6	33.0	35.7	36.9	39.5
18 : 8	224	-1.33274	29.4044	0.10524	23.8	24.7	25.2	26.6	27.5	29.4	31.7	33.1	35.8	37.0	39.5
18 : 9	225	-1.32869	29.4569	0.10516	23.8	24.7	25.2	26.6	27.5	29.5	31.7	33.1	35.9	37.1	39.6
18 : 10	226	-1.32369	29.5082	0.10508	23.9	24.8	25.3	26.7	27.6	29.5	31.8	33.2	35.9	37.1	39.6
18 : 11	227	-1.31773	29.5584	0.10501	23.9	24.8	25.3	26.7	27.6	29.6	31.8	33.2	36.0	37.2	39.7
19 : 0	228	-1.31080	29.6074	0.10495	24.0	24.8	25.3	26.7	27.7	29.6	31.9	33.3	36.0	37.2	39.7

Mid-upper arm circumference-for-age GIRLS (5 to 19 years)

5 to 19 years					Percentiles (MUAC in cm)										
Year : Month	Months	L	M	S	1st	3rd	5th	15th	25th	50th	75th	85th	95th	97th	99th
5 : 0	60	-0.49271	16.8168	0.09338	13.7	14.2	14.5	15.3	15.8	16.8	17.9	18.6	19.7	20.2	21.2
5 : 1	61	-0.50464	16.8623	0.09380	13.7	14.2	14.5	15.3	15.8	16.9	18.0	18.6	19.8	20.3	21.2
5 : 2	62	-0.51656	16.9078	0.09423	13.7	14.3	14.6	15.4	15.9	16.9	18.0	18.7	19.9	20.4	21.3
5 : 3	63	-0.52844	16.9532	0.09467	13.8	14.3	14.6	15.4	15.9	17.0	18.1	18.7	19.9	20.4	21.4
5 : 4	64	-0.54030	16.9986	0.09512	13.8	14.3	14.6	15.4	16.0	17.0	18.1	18.8	20.0	20.5	21.5
5 : 5	65	-0.55212	17.0440	0.09557	13.8	14.4	14.7	15.5	16.0	17.0	18.2	18.9	20.1	20.6	21.6
5 : 6	66	-0.56392	17.0895	0.09603	13.8	14.4	14.7	15.5	16.0	17.1	18.3	18.9	20.2	20.7	21.7
5 : 7	67	-0.57568	17.1350	0.09649	13.9	14.4	14.7	15.5	16.1	17.1	18.3	19.0	20.2	20.8	21.8
5 : 8	68	-0.58740	17.1807	0.09696	13.9	14.4	14.8	15.6	16.1	17.2	18.4	19.1	20.3	20.8	21.9
5 : 9	69	-0.59908	17.2265	0.09744	13.9	14.5	14.8	15.6	16.2	17.2	18.4	19.1	20.4	20.9	22.0
5 : 10	70	-0.61073	17.2725	0.09793	14.0	14.5	14.8	15.7	16.2	17.3	18.5	19.2	20.5	21.0	22.1
5 : 11	71	-0.62233	17.3188	0.09842	14.0	14.5	14.8	15.7	16.2	17.3	18.5	19.2	20.5	21.1	22.2
6 : 0	72	-0.63388	17.3653	0.09892	14.0	14.6	14.9	15.7	16.3	17.4	18.6	19.3	20.6	21.2	22.3
6 : 1	73	-0.64539	17.4122	0.09943	14.0	14.6	14.9	15.8	16.3	17.4	18.6	19.4	20.7	21.3	22.4
6 : 2	74	-0.65684	17.4594	0.09995	14.1	14.6	14.9	15.8	16.3	17.5	18.7	19.4	20.8	21.3	22.5
6 : 3	75	-0.66825	17.5071	0.10047	14.1	14.7	15.0	15.8	16.4	17.5	18.8	19.5	20.9	21.4	22.6
6 : 4	76	-0.67959	17.5551	0.10100	14.1	14.7	15.0	15.9	16.4	17.6	18.8	19.6	20.9	21.5	22.7
6 : 5	77	-0.69089	17.6037	0.10154	14.1	14.7	15.0	15.9	16.5	17.6	18.9	19.6	21.0	21.6	22.8
6 : 6	78	-0.70212	17.6528	0.10208	14.2	14.7	15.1	15.9	16.5	17.7	18.9	19.7	21.1	21.7	22.9
6 : 7	79	-0.71329	17.7024	0.10263	14.2	14.8	15.1	16.0	16.5	17.7	19.0	19.8	21.2	21.8	23.0
6 : 8	80	-0.72439	17.7527	0.10319	14.2	14.8	15.1	16.0	16.6	17.8	19.1	19.8	21.3	21.9	23.1
6 : 9	81	-0.73543	17.8036	0.10375	14.3	14.8	15.2	16.1	16.6	17.8	19.1	19.9	21.4	22.0	23.2
6 : 10	82	-0.74641	17.8551	0.10433	14.3	14.9	15.2	16.1	16.7	17.9	19.2	20.0	21.5	22.1	23.3
6 : 11	83	-0.75731	17.9073	0.10490	14.3	14.9	15.2	16.1	16.7	17.9	19.3	20.1	21.5	22.2	23.5
7 : 0	84	-0.76814	17.9602	0.10549	14.3	14.9	15.3	16.2	16.8	18.0	19.3	20.1	21.6	22.3	23.6
7 : 1	85	-0.77891	18.0138	0.10608	14.4	15.0	15.3	16.2	16.8	18.0	19.4	20.2	21.7	22.4	23.7
7 : 2	86	-0.78960	18.0680	0.10667	14.4	15.0	15.3	16.3	16.8	18.1	19.5	20.3	21.8	22.5	23.8
7 : 3	87	-0.80022	18.1230	0.10727	14.4	15.0	15.4	16.3	16.9	18.1	19.5	20.4	21.9	22.6	23.9
7 : 4	88	-0.81076	18.1787	0.10788	14.5	15.1	15.4	16.3	16.9	18.2	19.6	20.4	22.0	22.7	24.1
7 : 5	89	-0.82123	18.2351	0.10848	14.5	15.1	15.4	16.4	17.0	18.2	19.7	20.5	22.1	22.8	24.2
7 : 6	90	-0.83162	18.2923	0.10909	14.5	15.1	15.5	16.4	17.0	18.3	19.7	20.6	22.2	22.9	24.3
7 : 7	91	-0.84193	18.3502	0.10971	14.6	15.2	15.5	16.5	17.1	18.4	19.8	20.7	22.3	23.0	24.5
7 : 8	92	-0.85217	18.4088	0.11033	14.6	15.2	15.6	16.5	17.1	18.4	19.9	20.8	22.4	23.1	24.6
7 : 9	93	-0.86233	18.4683	0.11094	14.6	15.2	15.6	16.6	17.2	18.5	20.0	20.8	22.5	23.2	24.7
7 : 10	94	-0.87240	18.5285	0.11157	14.7	15.3	15.6	16.6	17.2	18.5	20.0	20.9	22.6	23.4	24.9
7 : 11	95	-0.88239	18.5895	0.11219	14.7	15.3	15.7	16.6	17.3	18.6	20.1	21.0	22.7	23.5	25.0
8 : 0	96	-0.89230	18.6514	0.11281	14.7	15.4	15.7	16.7	17.3	18.7	20.2	21.1	22.8	23.6	25.2
8 : 1	97	-0.90213	18.7140	0.11343	14.8	15.4	15.7	16.7	17.4	18.7	20.3	21.2	23.0	23.7	25.3
8 : 2	98	-0.91187	18.7773	0.11405	14.8	15.4	15.8	16.8	17.4	18.8	20.3	21.3	23.1	23.8	25.4
8 : 3	99	-0.92153	18.8415	0.11467	14.8	15.5	15.8	16.8	17.5	18.8	20.4	21.4	23.2	24.0	25.6
8 : 4	100	-0.93111	18.9063	0.11529	14.9	15.5	15.9	16.9	17.5	18.9	20.5	21.5	23.3	24.1	25.7
8 : 5	101	-0.94061	18.9719	0.11590	14.9	15.6	15.9	16.9	17.6	19.0	20.6	21.5	23.4	24.2	25.9
8 : 6	102	-0.95002	19.0382	0.11651	15.0	15.6	16.0	17.0	17.6	19.0	20.7	21.6	23.5	24.3	26.0
8 : 7	103	-0.95935	19.1052	0.11712	15.0	15.6	16.0	17.0	17.7	19.1	20.7	21.7	23.6	24.5	26.2
8 : 8	104	-0.96860	19.1729	0.11772	15.0	15.7	16.1	17.1	17.8	19.2	20.8	21.8	23.8	24.6	26.4
8 : 9	105	-0.97776	19.2412	0.11831	15.1	15.7	16.1	17.1	17.8	19.2	20.9	21.9	23.9	24.7	26.5
8 : 10	106	-0.98685	19.3102	0.11890	15.1	15.8	16.1	17.2	17.9	19.3	21.0	22.0	24.0	24.9	26.7
8 : 11	107	-0.99585	19.3799	0.11948	15.2	15.8	16.2	17.2	17.9	19.4	21.1	22.1	24.1	25.0	26.8
9 : 0	108	-1.00477	19.4501	0.12006	15.2	15.9	16.2	17.3	18.0	19.5	21.2	22.2	24.2	25.1	27.0
9 : 1	109	-1.01360	19.5210	0.12062	15.2	15.9	16.3	17.4	18.1	19.5	21.2	22.3	24.4	25.3	27.2
9 : 2	110	-1.02236	19.5924	0.12117	15.3	16.0	16.3	17.4	18.1	19.6	21.3	22.4	24.5	25.4	27.3
9 : 3	111	-1.03103	19.6645	0.12171	15.3	16.0	16.4	17.5	18.2	19.7	21.4	22.5	24.6	25.5	27.5
9 : 4	112	-1.03963	19.7371	0.12224	15.4	16.1	16.4	17.5	18.2	19.7	21.5	22.6	24.7	25.7	27.6
9 : 5	113	-1.04814	19.8104	0.12276	15.4	16.1	16.5	17.6	18.3	19.8	21.6	22.7	24.9	25.8	27.8
9 : 6	114	-1.05657	19.8842	0.12327	15.5	16.2	16.5	17.6	18.4	19.9	21.7	22.8	25.0	25.9	28.0
9 : 7	115	-1.06493	19.9586	0.12376	15.5	16.2	16.6	17.7	18.4	20.0	21.8	22.9	25.1	26.1	28.1
9 : 8	116	-1.07321	20.0336	0.12424	15.6	16.3	16.7	17.8	18.5	20.0	21.9	23.0	25.2	26.2	28.3
9 : 9	117	-1.08141	20.1093	0.12471	15.6	16.3	16.7	17.8	18.6	20.1	22.0	23.1	25.4	26.4	28.5

5 to 19 years					Percentiles (MUAC in cm)										
Year : Month	Months	L	M	S	1st	3rd	5th	15th	25th	50th	75th	85th	95th	97th	99th
9 : 10	118	-1.08954	20.1855	0.12515	15.7	16.4	16.8	17.9	18.6	20.2	22.1	23.2	25.5	26.5	28.6
9 : 11	119	-1.09759	20.2623	0.12559	15.7	16.4	16.8	17.9	18.7	20.3	22.1	23.3	25.6	26.6	28.8
10 : 0	120	-1.10557	20.3398	0.12601	15.8	16.5	16.9	18.0	18.8	20.3	22.2	23.4	25.7	26.8	29.0
10 : 1	121	-1.11347	20.4178	0.12641	15.8	16.5	16.9	18.1	18.8	20.4	22.3	23.5	25.9	26.9	29.2
10 : 2	122	-1.12131	20.4965	0.12679	15.9	16.6	17.0	18.1	18.9	20.5	22.4	23.6	26.0	27.1	29.3
10 : 3	123	-1.12907	20.5758	0.12716	15.9	16.6	17.1	18.2	19.0	20.6	22.5	23.7	26.1	27.2	29.5
10 : 4	124	-1.13676	20.6557	0.12751	16.0	16.7	17.1	18.3	19.0	20.7	22.6	23.8	26.2	27.3	29.7
10 : 5	125	-1.14439	20.7362	0.12783	16.1	16.8	17.2	18.3	19.1	20.7	22.7	23.9	26.4	27.5	29.8
10 : 6	126	-1.15194	20.8172	0.12814	16.1	16.8	17.2	18.4	19.2	20.8	22.8	24.0	26.5	27.6	30.0
10 : 7	127	-1.15943	20.8989	0.12843	16.2	16.9	17.3	18.5	19.2	20.9	22.9	24.1	26.6	27.7	30.2
10 : 8	128	-1.16685	20.9810	0.12870	16.2	17.0	17.4	18.5	19.3	21.0	23.0	24.3	26.8	27.9	30.3
10 : 9	129	-1.17420	21.0637	0.12895	16.3	17.0	17.4	18.6	19.4	21.1	23.1	24.4	26.9	28.0	30.5
10 : 10	130	-1.18149	21.1468	0.12918	16.4	17.1	17.5	18.7	19.5	21.1	23.2	24.5	27.0	28.2	30.7
10 : 11	131	-1.18871	21.2304	0.12939	16.4	17.1	17.6	18.7	19.5	21.2	23.3	24.6	27.1	28.3	30.8
11 : 0	132	-1.19587	21.3144	0.12958	16.5	17.2	17.6	18.8	19.6	21.3	23.4	24.7	27.3	28.4	31.0
11 : 1	133	-1.20297	21.3988	0.12976	16.5	17.3	17.7	18.9	19.7	21.4	23.5	24.8	27.4	28.6	31.1
11 : 2	134	-1.21000	21.4835	0.12991	16.6	17.3	17.8	19.0	19.8	21.5	23.6	24.9	27.5	28.7	31.3
11 : 3	135	-1.21697	21.5686	0.13004	16.7	17.4	17.8	19.0	19.8	21.6	23.7	25.0	27.6	28.8	31.5
11 : 4	136	-1.22389	21.6540	0.13016	16.7	17.5	17.9	19.1	19.9	21.7	23.8	25.1	27.8	29.0	31.6
11 : 5	137	-1.23074	21.7397	0.13025	16.8	17.5	18.0	19.2	20.0	21.7	23.9	25.2	27.9	29.1	31.8
11 : 6	138	-1.23753	21.8256	0.13032	16.9	17.6	18.0	19.3	20.1	21.8	24.0	25.3	28.0	29.2	31.9
11 : 7	139	-1.24427	21.9117	0.13038	16.9	17.7	18.1	19.3	20.2	21.9	24.0	25.4	28.1	29.4	32.1
11 : 8	140	-1.25095	21.9980	0.13041	17.0	17.8	18.2	19.4	20.2	22.0	24.1	25.5	28.2	29.5	32.2
11 : 9	141	-1.25757	22.0845	0.13043	17.1	17.8	18.3	19.5	20.3	22.1	24.2	25.6	28.4	29.6	32.4
11 : 10	142	-1.26414	22.1710	0.13043	17.1	17.9	18.3	19.6	20.4	22.2	24.3	25.7	28.5	29.7	32.5
11 : 11	143	-1.27064	22.2574	0.13041	17.2	18.0	18.4	19.6	20.5	22.3	24.4	25.8	28.6	29.9	32.6
12 : 0	144	-1.27709	22.3438	0.13037	17.3	18.1	18.5	19.7	20.6	22.3	24.5	25.9	28.7	30.0	32.8
12 : 1	145	-1.28349	22.4300	0.13032	17.4	18.1	18.6	19.8	20.6	22.4	24.6	26.0	28.8	30.1	32.9
12 : 2	146	-1.28983	22.5161	0.13025	17.4	18.2	18.6	19.9	20.7	22.5	24.7	26.1	28.9	30.2	33.1
12 : 3	147	-1.29611	22.6018	0.13017	17.5	18.3	18.7	20.0	20.8	22.6	24.8	26.2	29.0	30.3	33.2
12 : 4	148	-1.30233	22.6872	0.13008	17.6	18.3	18.8	20.0	20.9	22.7	24.9	26.3	29.2	30.5	33.3
12 : 5	149	-1.30850	22.7722	0.12997	17.7	18.4	18.9	20.1	21.0	22.8	25.0	26.4	29.3	30.6	33.5
12 : 6	150	-1.31461	22.8568	0.12985	17.7	18.5	18.9	20.2	21.0	22.9	25.1	26.5	29.4	30.7	33.6
12 : 7	151	-1.32067	22.9408	0.12971	17.8	18.6	19.0	20.3	21.1	22.9	25.2	26.6	29.5	30.8	33.7
12 : 8	152	-1.32667	23.0242	0.12957	17.9	18.6	19.1	20.3	21.2	23.0	25.3	26.7	29.6	30.9	33.8
12 : 9	153	-1.33261	23.1069	0.12941	17.9	18.7	19.2	20.4	21.3	23.1	25.4	26.8	29.7	31.0	33.9
12 : 10	154	-1.33850	23.1889	0.12925	18.0	18.8	19.2	20.5	21.4	23.2	25.4	26.9	29.8	31.1	34.1
12 : 11	155	-1.34434	23.2701	0.12907	18.1	18.9	19.3	20.6	21.4	23.3	25.5	27.0	29.9	31.2	34.2
13 : 0	156	-1.35012	23.3505	0.12889	18.2	18.9	19.4	20.7	21.5	23.4	25.6	27.1	30.0	31.3	34.3
13 : 1	157	-1.35584	23.4301	0.12870	18.2	19.0	19.5	20.7	21.6	23.4	25.7	27.1	30.1	31.4	34.4
13 : 2	158	-1.36150	23.5087	0.12850	18.3	19.1	19.5	20.8	21.7	23.5	25.8	27.2	30.2	31.5	34.5
13 : 3	159	-1.36711	23.5865	0.12830	18.4	19.1	19.6	20.9	21.7	23.6	25.9	27.3	30.3	31.6	34.6
13 : 4	160	-1.37265	23.6632	0.12809	18.4	19.2	19.7	20.9	21.8	23.7	25.9	27.4	30.3	31.7	34.7
13 : 5	161	-1.37814	23.7390	0.12787	18.5	19.3	19.7	21.0	21.9	23.7	26.0	27.5	30.4	31.8	34.8
13 : 6	162	-1.38357	23.8137	0.12766	18.6	19.4	19.8	21.1	22.0	23.8	26.1	27.6	30.5	31.9	34.9
13 : 7	163	-1.38894	23.8873	0.12744	18.6	19.4	19.9	21.2	22.0	23.9	26.2	27.6	30.6	32.0	35.0
13 : 8	164	-1.39425	23.9598	0.12721	18.7	19.5	19.9	21.2	22.1	24.0	26.3	27.7	30.7	32.1	35.1
13 : 9	165	-1.39950	24.0312	0.12699	18.8	19.6	20.0	21.3	22.2	24.0	26.3	27.8	30.8	32.1	35.2
13 : 10	166	-1.40469	24.1013	0.12676	18.8	19.6	20.1	21.4	22.2	24.1	26.4	27.9	30.8	32.2	35.3
13 : 11	167	-1.40982	24.1703	0.12653	18.9	19.7	20.1	21.4	22.3	24.2	26.5	27.9	30.9	32.3	35.4
14 : 0	168	-1.41489	24.2379	0.12630	19.0	19.7	20.2	21.5	22.4	24.2	26.5	28.0	31.0	32.4	35.4
14 : 1	169	-1.41989	24.3043	0.12608	19.0	19.8	20.3	21.6	22.4	24.3	26.6	28.1	31.1	32.5	35.5
14 : 2	170	-1.42484	24.3693	0.12585	19.1	19.9	20.3	21.6	22.5	24.4	26.7	28.2	31.1	32.5	35.6
14 : 3	171	-1.42971	24.4330	0.12563	19.1	19.9	20.4	21.7	22.6	24.4	26.7	28.2	31.2	32.6	35.7
14 : 4	172	-1.43453	24.4954	0.12541	19.2	20.0	20.4	21.7	22.6	24.5	26.8	28.3	31.3	32.7	35.7
14 : 5	173	-1.43928	24.5565	0.12519	19.3	20.0	20.5	21.8	22.7	24.6	26.9	28.3	31.4	32.7	35.8
14 : 6	174	-1.44397	24.6163	0.12498	19.3	20.1	20.6	21.9	22.7	24.6	26.9	28.4	31.4	32.8	35.9
14 : 7	175	-1.44859	24.6747	0.12476	19.4	20.2	20.6	21.9	22.8	24.7	27.0	28.5	31.5	32.9	36.0
14 : 8	176	-1.45315	24.7318	0.12456	19.4	20.2	20.7	22.0	22.8	24.7	27.0	28.5	31.5	32.9	36.0

5 to 19 years					Percentiles (MUAC in cm)										
Year : Month	Months	L	M	S	1st	3rd	5th	15th	25th	50th	75th	85th	95th	97th	99th
14 : 9	177	-1.45764	24.7876	0.12436	19.5	20.3	20.7	22.0	22.9	24.8	27.1	28.6	31.6	33.0	36.1
14 : 10	178	-1.46207	24.8421	0.12416	19.5	20.3	20.8	22.1	23.0	24.8	27.2	28.6	31.7	33.1	36.2
14 : 11	179	-1.46642	24.8952	0.12397	19.6	20.4	20.8	22.1	23.0	24.9	27.2	28.7	31.7	33.1	36.2
15 : 0	180	-1.47072	24.9470	0.12378	19.6	20.4	20.9	22.2	23.1	24.9	27.3	28.8	31.8	33.2	36.3
15 : 1	181	-1.47494	24.9975	0.12360	19.7	20.5	20.9	22.2	23.1	25.0	27.3	28.8	31.8	33.2	36.3
15 : 2	182	-1.47910	25.0466	0.12343	19.7	20.5	21.0	22.3	23.2	25.0	27.4	28.9	31.9	33.3	36.4
15 : 3	183	-1.48319	25.0944	0.12326	19.8	20.6	21.0	22.3	23.2	25.1	27.4	28.9	31.9	33.3	36.5
15 : 4	184	-1.48721	25.1409	0.12311	19.8	20.6	21.1	22.4	23.2	25.1	27.5	29.0	32.0	33.4	36.5
15 : 5	185	-1.49116	25.1861	0.12296	19.8	20.6	21.1	22.4	23.3	25.2	27.5	29.0	32.0	33.4	36.6
15 : 6	186	-1.49504	25.2299	0.12281	19.9	20.7	21.1	22.5	23.3	25.2	27.6	29.1	32.1	33.5	36.6
15 : 7	187	-1.49886	25.2725	0.12268	19.9	20.7	21.2	22.5	23.4	25.3	27.6	29.1	32.1	33.5	36.7
15 : 8	188	-1.50260	25.3139	0.12255	20.0	20.8	21.2	22.5	23.4	25.3	27.6	29.1	32.2	33.6	36.7
15 : 9	189	-1.50628	25.3541	0.12243	20.0	20.8	21.3	22.6	23.5	25.4	27.7	29.2	32.2	33.6	36.8
15 : 10	190	-1.50989	25.3931	0.12232	20.0	20.8	21.3	22.6	23.5	25.4	27.7	29.2	32.3	33.7	36.8
15 : 11	191	-1.51343	25.4309	0.12222	20.1	20.9	21.3	22.6	23.5	25.4	27.8	29.3	32.3	33.7	36.9
16 : 0	192	-1.51691	25.4676	0.12213	20.1	20.9	21.4	22.7	23.6	25.5	27.8	29.3	32.4	33.8	36.9
16 : 1	193	-1.52031	25.5032	0.12204	20.1	20.9	21.4	22.7	23.6	25.5	27.8	29.3	32.4	33.8	37.0
16 : 2	194	-1.52364	25.5377	0.12196	20.2	21.0	21.4	22.8	23.6	25.5	27.9	29.4	32.4	33.9	37.0
16 : 3	195	-1.52691	25.5711	0.12189	20.2	21.0	21.5	22.8	23.7	25.6	27.9	29.4	32.5	33.9	37.1
16 : 4	196	-1.53011	25.6035	0.12183	20.2	21.0	21.5	22.8	23.7	25.6	28.0	29.5	32.5	34.0	37.1
16 : 5	197	-1.53324	25.6348	0.12177	20.3	21.1	21.5	22.8	23.7	25.6	28.0	29.5	32.6	34.0	37.2
16 : 6	198	-1.53630	25.6652	0.12173	20.3	21.1	21.6	22.9	23.8	25.7	28.0	29.5	32.6	34.0	37.2
16 : 7	199	-1.53930	25.6946	0.12169	20.3	21.1	21.6	22.9	23.8	25.7	28.0	29.6	32.6	34.1	37.3
16 : 8	200	-1.54222	25.7231	0.12166	20.3	21.1	21.6	22.9	23.8	25.7	28.1	29.6	32.7	34.1	37.3
16 : 9	201	-1.54508	25.7507	0.12164	20.4	21.2	21.6	23.0	23.8	25.8	28.1	29.6	32.7	34.2	37.4
16 : 10	202	-1.54788	25.7774	0.12163	20.4	21.2	21.7	23.0	23.9	25.8	28.1	29.7	32.8	34.2	37.4
16 : 11	203	-1.55061	25.8033	0.12162	20.4	21.2	21.7	23.0	23.9	25.8	28.2	29.7	32.8	34.2	37.4
17 : 0	204	-1.55328	25.8284	0.12162	20.4	21.2	21.7	23.0	23.9	25.8	28.2	29.7	32.8	34.3	37.5
17 : 1	205	-1.55588	25.8527	0.12163	20.4	21.3	21.7	23.0	23.9	25.9	28.2	29.7	32.9	34.3	37.5
17 : 2	206	-1.55843	25.8763	0.12165	20.5	21.3	21.7	23.1	24.0	25.9	28.2	29.8	32.9	34.3	37.6
17 : 3	207	-1.56091	25.8991	0.12167	20.5	21.3	21.8	23.1	24.0	25.9	28.3	29.8	32.9	34.4	37.6
17 : 4	208	-1.56333	25.9213	0.12170	20.5	21.3	21.8	23.1	24.0	25.9	28.3	29.8	33.0	34.4	37.7
17 : 5	209	-1.56569	25.9429	0.12174	20.5	21.3	21.8	23.1	24.0	25.9	28.3	29.9	33.0	34.4	37.7
17 : 6	210	-1.56799	25.9638	0.12178	20.5	21.3	21.8	23.1	24.0	26.0	28.3	29.9	33.0	34.5	37.8
17 : 7	211	-1.57024	25.9842	0.12183	20.6	21.4	21.8	23.2	24.1	26.0	28.4	29.9	33.1	34.5	37.8
17 : 8	212	-1.57243	26.0040	0.12189	20.6	21.4	21.8	23.2	24.1	26.0	28.4	29.9	33.1	34.6	37.8
17 : 9	213	-1.57456	26.0233	0.12195	20.6	21.4	21.9	23.2	24.1	26.0	28.4	30.0	33.1	34.6	37.9
17 : 10	214	-1.57664	26.0421	0.12202	20.6	21.4	21.9	23.2	24.1	26.0	28.4	30.0	33.2	34.6	37.9
17 : 11	215	-1.57867	26.0605	0.12210	20.6	21.4	21.9	23.2	24.1	26.1	28.5	30.0	33.2	34.7	38.0
18 : 0	216	-1.58064	26.0784	0.12218	20.6	21.4	21.9	23.2	24.1	26.1	28.5	30.0	33.2	34.7	38.0
18 : 1	217	-1.58256	26.0958	0.12227	20.6	21.4	21.9	23.3	24.2	26.1	28.5	30.1	33.2	34.7	38.1
18 : 2	218	-1.58443	26.1128	0.12236	20.6	21.5	21.9	23.3	24.2	26.1	28.5	30.1	33.3	34.8	38.1
18 : 3	219	-1.58625	26.1295	0.12246	20.7	21.5	21.9	23.3	24.2	26.1	28.5	30.1	33.3	34.8	38.2
18 : 4	220	-1.58803	26.1457	0.12256	20.7	21.5	22.0	23.3	24.2	26.1	28.6	30.1	33.3	34.8	38.2
18 : 5	221	-1.58975	26.1616	0.12267	20.7	21.5	22.0	23.3	24.2	26.2	28.6	30.2	33.4	34.9	38.3
18 : 6	222	-1.59143	26.1772	0.12278	20.7	21.5	22.0	23.3	24.2	26.2	28.6	30.2	33.4	34.9	38.3
18 : 7	223	-1.59307	26.1924	0.12290	20.7	21.5	22.0	23.3	24.2	26.2	28.6	30.2	33.4	34.9	38.4
18 : 8	224	-1.59466	26.2073	0.12302	20.7	21.5	22.0	23.3	24.2	26.2	28.6	30.2	33.5	35.0	38.4
18 : 9	225	-1.59621	26.2220	0.12315	20.7	21.5	22.0	23.3	24.3	26.2	28.7	30.2	33.5	35.0	38.5
18 : 10	226	-1.59771	26.2363	0.12328	20.7	21.5	22.0	23.4	24.3	26.2	28.7	30.3	33.5	35.1	38.5
18 : 11	227	-1.59918	26.2505	0.12342	20.7	21.5	22.0	23.4	24.3	26.3	28.7	30.3	33.6	35.1	38.5
19 : 0	228	-1.60061	26.2644	0.12356	20.7	21.6	22.0	23.4	24.3	26.3	28.7	30.3	33.6	35.1	38.6
