# Supplementary figures and images for: A growth reference for mid upper arm circumference for age among school age children and adolescents, and validation for mortality: growth curve construction and longitudinal cohort study
Source: BMJ. 2017 Aug 3;358:j3423. doi: 10.1136/bmj.j3423 (PMC5541507; doi:10.1136/bmj.j3423)

# Girls

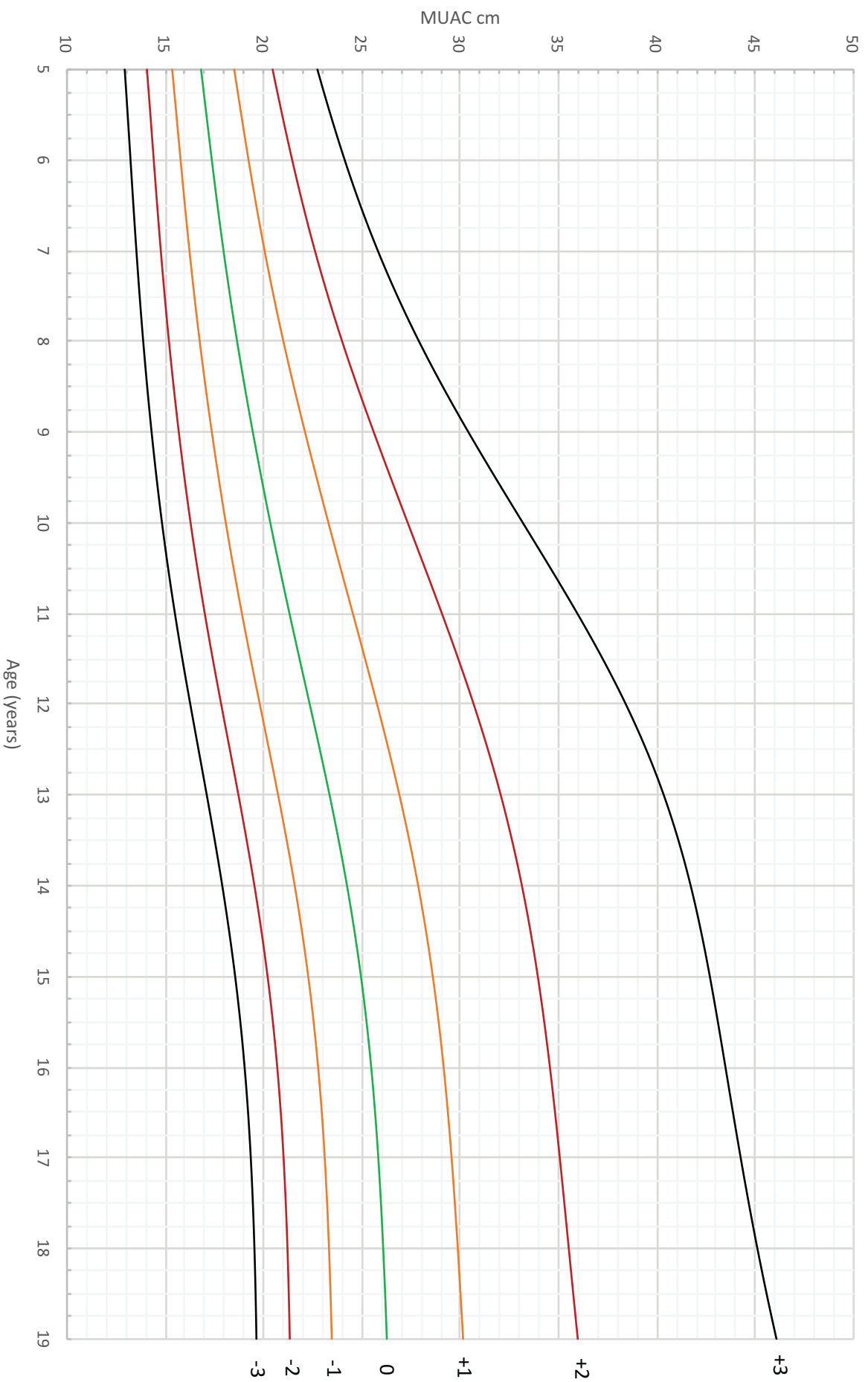

Supplement: Supplementary file 2 — Supplementary figure: MUAC-for-age chart in girls for clinical and programmatic use [file mral036206.wf1.pdf]

# Boys

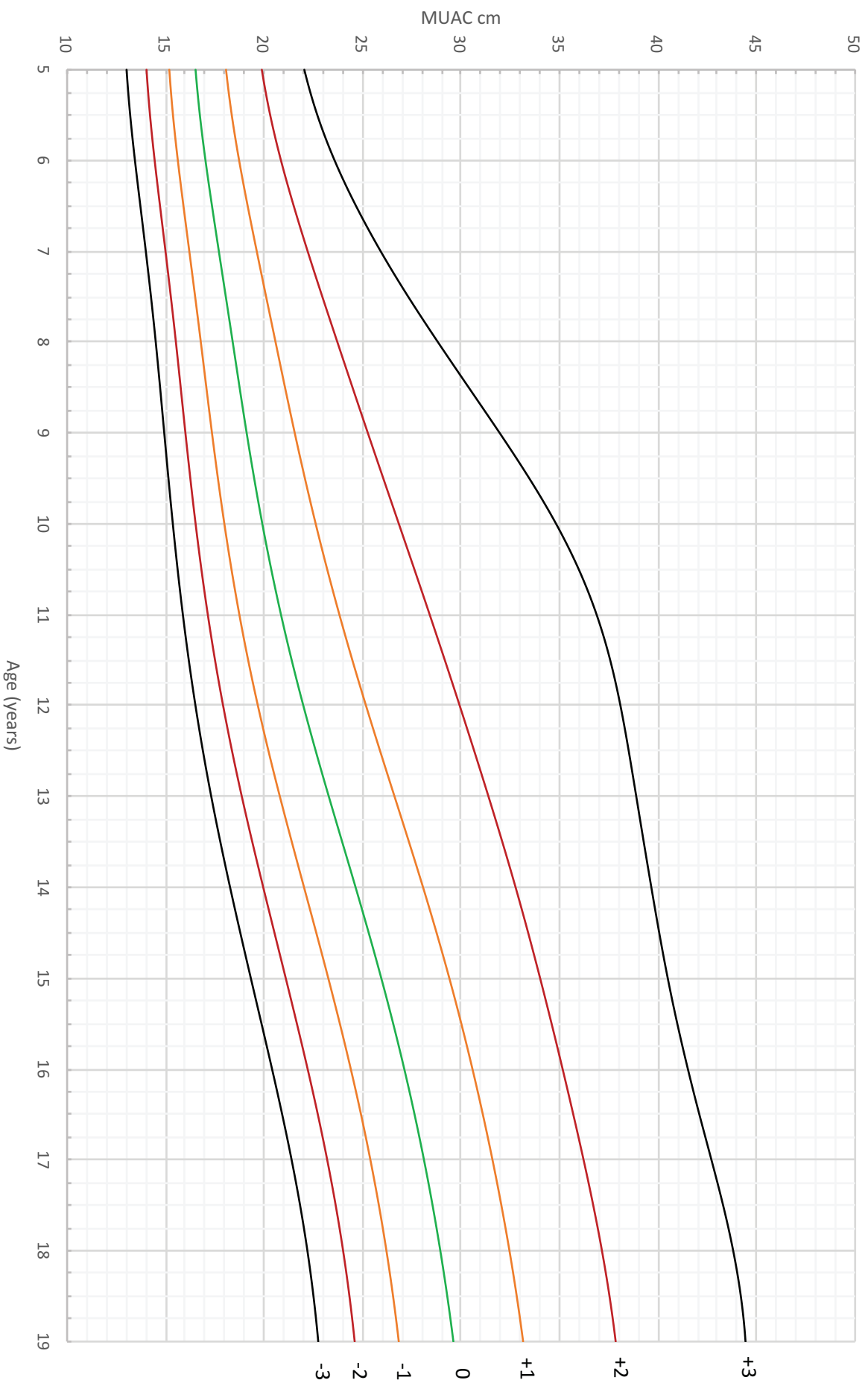

Supplement: Supplementary file 3 — Supplementary figure: MUAC-for-age chart in boys for clinical and programmatic use [file mral036206.wf2.pdf]
